# Assessing the female urogenital-rectal axis microbiome: focus on endometriosis and recurrent implantation failure

**DOI:** 10.1186/s12958-026-01595-0

**Published:** 2026-07-21

**Authors:** Bárbara A. Folch, Inmaculada Pérez-Prieto, Eduardo Salas-Espejo, Alberto Sola-Leyva, Analuce Canha-Gouveia, Nerea M. Molina, Eva Vargas, Irene Leonés-Baños, Miriam Gámiz-Aguilera, Celia M. Tenorio, María José Sáez-Lara, Susana Ruíz-Durán, Rocío Sánchez-Ruíz, Bárbara Romero, José Antonio Castilla, Signe Altmäe

**Affiliations:** 1 Department of Biochemistry and Molecular Biology, Faculty of Sciences, University of Granada, Granada, Spain; 2 Instituto de Investigación Biosanitaria ibs.GRANADA, Granada, Spain; 3 Celvia CC AS, Tartu, 50410 Estonia; 4 Division of Obstetrics and Gynaecology, Department of Clinical Science, Intervention and Technology, Karolinska Institutet, Huddinge, Sweden; 5 Department of Gynaecology and Reproductive Medicine, Karolinska University Hospital, Huddinge, Sweden; 6 Department of Obstetrics and Gynaecology, Institute of Clinical Medicine, University of Tartu, Tartu, Estonia; 7 Department of Anatomy and Cell Biology, Faculty of Medicine, University of Cantabria, Santander, Spain; 8 Department of Philosophy I, Faculty of Philosophy, Cátedra Youngner de Bioética Empírica, FiloLab-UGR, University of Granada, Granada, Spain; 9 Systems Biology Unit, Department of Experimental Biology, Faculty of Experimental Sciences, University of Jaen, Jaen, Spain; 10 Hospital Universitario Virgen de las Nieves UGC de Obstetricia y Ginecología, Granada, Spain; 11 Hospital Universitario Virgen de las Nieves UGC de Obstetricia y Ginecología, Unidad de Reproducción, Granada, Spain; 12 Department of Human Anatomy and Embryology, Faculty of Medicine, University of Granada, Granada, Spain

**Keywords:** 16S rRNA gene sequencing, Endometriosis, Female infertility, Microbiome, Microbiota, Recurrent implantation failure

## Abstract

**Background:**

Emerging evidence suggests microbial dysbiosis may contribute to gynaecological pathologies, including endometriosis and recurrent implantation failure (RIF). The vaginal microbiome is well-characterised, with *Lactobacillus*-dominance as a hallmark of health, yet interactions between vaginal, cervical, and endometrial microbiomes remain inconclusive. The adjacent sites to the vagina, rectal and urinary environments in infertile women are scarcely studied.

**Methods:**

This cross-sectional study included 136 reproductive-aged women, enrolled at the Reproductive Unit of the University Hospital Virgen de las Nieves (Granada, Spain) between March 2019 and March 2024. Each participant provided five samples during the mid-secretory phase: vaginal and cervical swabs, endometrial brushing, urine, and rectal swabs. The microbiome was analysed using 16S rRNA gene sequencing (V4 region) with functional profiles inferred using PICRUSt2. Diversity and bacterial abundance were compared between endometriosis, RIF, and controls, adjusting for age, body mass index, and antimicrobial use.

**Results:**

In total, 520 samples from 104 women were analysed and revealed shared microbial profiles across the vagina, cervix, endometrium, and urine. The rectal microbiome differed significantly from urogenital sites (Global PERMANOVA, adj *p*-value = 0.001, R^2^ = 0.259). A *Lactobacillus* gradient in the reproductive tract was observed, with dominance > 98% in the lower tract, 69.72% in urine, and 46.25% in the endometrium. Moreover, *Lactobacillus* negatively correlated with all other genera within the reproductive tract. Significant differential abundance was detected between body sites: 15 bacteria between vagina-cervix, 166 vagina-uterus and 172 cervix-uterus (all adj *p*-values < 0.05). Diversity comparisons between the condition groups (endometriosis and RIF) and controls at each anatomical sites revealed no significant differences in microbial communities and functional pathways. However, four bacterial genera showed a significantly different abundance between the endometriosis and controls in the vagina.

**Conclusions:**

Our study results provide knowledge about the microbial composition throughout the female urogenital tract and rectum, highlighting the interindividual variability rather than the site-specificity. The vaginal bacteria that associated with endometriosis should be investigated further for clarifying their potential as non-invasive biomarkers of the disease. Microbiomes of other urogenital sites do not seem to associate with endometriosis, and microbiomes of the urogenital-rectal axis do not seem to correlate with RIF.

**Trial registration:**

N/A.

**Supplementary Information:**

The online version contains supplementary material available at 10.1186/s12958-026-01595-0.

## Introduction

The female reproductive tract harbours a large number of microorganisms that have been established as a continuum from the vagina to the upper reproductive tract [1–5]. The vaginal microbiome is considered a high bacterial biomass site whose composition and diversity have been extensively studied and characterised [6, 7]. Notably, a *Lactobacillus-*dominance of 90–95% has been associated with a good reproductive health [8, 9]. *Lactobacillus* acts as a ‘guardian’ by producing lactic acid, which contributes to the maintenance of vaginal pH around 4.5 and prevents the growth of pathogenic opportunistic bacteria [10–12]. The adjacent site, the cervix, also possesses *Lactobacillus*-dominance [13–18]; however, there is still limited consensus regarding its microbial composition. Some studies have reported the abundance of *Lactobacillus* genus around 50% followed by *Pseudomonas* and *Cloacibacterium* genera in cervical samples [17, 19], while others highlight the presence of different bacterial genera such as *Gardnerella*,* Veillonella*,* Prevotella*,* Sneathia*,* Fusobacterium*,* Streptococcus*,* Atopobium* or *Pseudomonas* [15, 17, 20]. Unlike the lower reproductive tract, the uterine cavity possesses low microbial biomass with a higher microbial diversity [21–24]. However, the presence of microbes in the uterus remains a subject of ongoing debate, due to the sampling difficulties [19, 25]. Different studies suggest the *Lactobacillus*-dominance in the endometrium, while other studies have detected dominance of other bacterial genera such as *Acinetobacter*,* Pseudomonas*,* Comamonadaceae*,* Bifidobacterium*,* Gardnerella*,* Prevotella* and *Streptococcus* [1, 2, 8, 19, 26–28]. The female reproductive tract is intricately interconnected, both directly and indirectly, with other physiological systems, such as the urinary and intestinal tracts [29, 30]. These sites anatomically adjacent to the reproductive tract (urinary and rectal environments) have been scarcely studied in terms of their microbial composition in infertile women. The urobiome, the microbial communities that harbour in the urinary tract, is considered a low bacterial biomass site that contains a wide range of bacteria [31–33], although most studies report a *Lactobacillus*-dominance [32, 34–37]. In the rectal microbiome, taxa belonging to the *Lactobacillus* genus, as well as the Eubacteria and Bacteroidetes groups, were identified [38–41]. Moreover, the rectal microbiome composition is believed to reflect the gut microbiome composition [40], which is gaining research interest in the reproductive field as the gut microbes seem to play important role in oestrogen metabolism [42].

Increasing evidence suggests that alterations in the microbial composition of the female reproductive tract and the gut can affect reproductive functions as well as contribute to the development of certain gynaecological pathologies [17]. Endometriosis is a chronic, oestrogen-dependent condition with an inflammatory nature, in which microbial dysbiosis could enhance the (pro)inflammatory environment [43] and lead to abnormal production of oestrogens [42, 44]. In women with recurrent implantation failure (RIF), which is suggested to involve an aberrant endometrial function, the microbial dysbiosis has been proposed as one factor contributing to the implantation failure [45, 46]. Nevertheless, there is no agreement in the core microbial composition of the female reproductive tract [47]. Further, the anatomically adjacent sites have been scarcely investigated, highlighting the need for well-controlled studies with larger sample sizes to better elucidate and understand the microbiome in female reproductive health and disease.

In the present study, we set out to map the microbiome along the female reproductive tract and its adjacent sites to identify the potential microbial similarities and interactions between these locations, and to characterise the microbial composition associated with common gynaecological disorders such as endometriosis and RIF.

## Materials and methods

### Study population

**Fig. 1:**
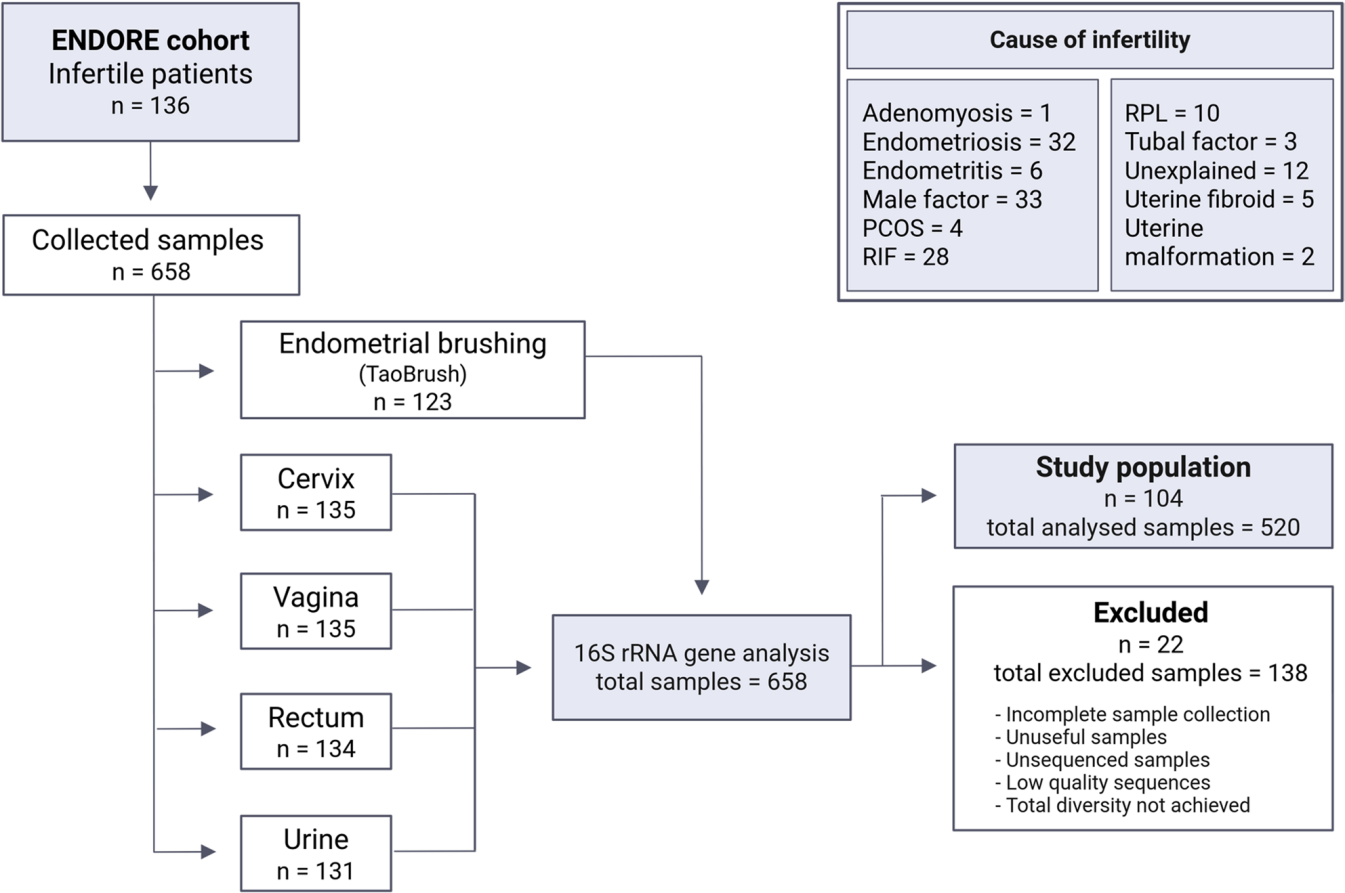
Flowchart of sample selection for the 16S rRNA gene analysis from the ENDORE cohort. We considered only the participants with all types of samples: vaginal, cervical and rectal swabs, endometrial brushing and urine samples. After the sequencing process, samples with low quality sequences and/or those that did not capture the total bacterial diversity were excluded. Finally, our study population consisted of 104 women. PCOS (polycystic ovary syndrome); RIF (recurrent implantation failure); RPL (recurrent pregnancy loss)

A total of 136 infertile women were recruited at the Reproductive Unit of the University Hospital Virgen de las Nieves (Granada, Spain) between March 2019 and March 2024. All participants had experienced at least one year of unprotected intercourse without conception. The inclusion criteria required women at natural cycle prior to infertility treatment. Infertility causes comprised adenomyosis, endometriosis, endometritis, male factor infertility, polycystic ovary syndrome (PCOS), RIF, recurrent pregnancy loss (RPL), tubal factor, unexplained infertility, uterine fibroids, and uterine malformations (Fig. 1). Endometriosis was confirmed via laparotomy or laparoscopic surgery with histological confirmation or ultrasound, while endometritis was identified histologically by the presence of plasma cells in the endometrial stroma. Male factor infertility was defined according to the World Health Organization (WHO) values for semen analysis [48]. RIF was defined as the failure to achieve pregnancy after the transfer of at least three good-quality embryos, and RPL was defined as the loss of two or more pregnancies. Finally, unexplained infertility was diagnosed when medical examinations revealed no reproductive complications in either partner. Exclusion criteria included age ≥ 43 years, gynaecological tumours, systemic diseases, pelvic inflammatory disease, or other pelvic pathological conditions. Anthropometric measurements were taken from each participant and information regarding the frequency of antibiotic, antiviral, or antifungal use during the three months preceding sample collection was recorded.

### Sample collection

All women exhibited natural menstrual cycles and had not received any form of hormonal treatment during the menstrual phase of the sample collection. Each participant self-monitored urinary luteinising hormone (LH) levels at to identify the LH surge, to allow the estimation of the menstrual cycle phase (Clearblue Digital Ovulation Test, Swiss Precision Diagnostics, Geneva, Switzerland). Samples were collected at the mid-secretory menstrual phase, 7–9 days after the LH peak. Five samples were taken sequentially from each woman: (i) mid-catch urine sample (stored in eNAT^®^ 608CS01R; COPAN Italia, Brescia); (ii) a vaginal swab (eNAT^®^ 606CS01R; COPAN Italia); (iii) a cervical swab (eNAT^®^ 606CS01L; COPAN Italia); (iv) an endometrial sample using Tao Brush IUMC endometrial sampler equipped with a protective sheath to minimise contamination from the lower reproductive tract (Cook Medical, Madrid, Spain; stored in eNAT^®^ 606 C; COPAN Italia); and (v) a rectal swab (eNAT^®^ 606CS01R; COPAN Italia). All samples were stored at -80 °C until further analysis. Additionally, sampling controls were collected, including environmental controls from the gynaecological practice setting, along with controls from the gloves used during sample collection, controls of COPAN stabilisation medium and a control of pliers used to cut the Tao Brush IUMC endometrial, which were included as negative controls in further analyses.

### DNA extraction and sequencing of 16S rRNA gene fragment

Microbial DNA was extracted from the samples using the Qiagen QIAamp UCP kit with Pathogen Lysis Tube S (Qiagen, Venlo, Netherlands) following the manufacturer’s instructions. DNA quality and purity (230/260/280 nm) was evaluated by NanoDrop ND-1000 Spectrophotometer (Thermo Scientific, Waltham, Massachusetts, USA) and DNA quantification was carried out by Qubit 4 fluorometer (Thermo Fisher Scientific). To assess potential microbial contamination, both negative and positive controls were incorporated and processed alongside the biological samples. Negative controls were included at multiple stages of the workflow, encompassing sample collection, DNA extraction, library preparation, and sequencing. In addition, positive controls based on the ZymoBIOMICS™ Microbial Community Standard (Zymo Research, Irvine, California, USA) were included.

The microbiome was profiled by amplifying the hypervariable region V4 using the 515F (5’-GTGYCAGCMGCCGCGGTAA-3’) and 806R (5’- GGACTACNVGGGTWTCTAAT-3’) primers. All PCR reactions were performed in a 25 µl total volume containing 12.5 µl 2X KAPA HiFi Hotstart ready mix (KAPA, Biosystems, Wilmington, MA, USA), 5 µl of each primer (1 mΜ), and 2.5 µl of extracted DNA under the following cycling conditions: initial denaturation at 95 °C for 3 min, followed by a cyclic 3-step stage consisting of 35 cycles of denaturation at 95 °C for 30 s, annealing at 55 °C for 30 s, and extension at 72 °C for 5 min. The resulting amplicons were tested by electrophoresis using 2% agarose gel, the size of each amplicon was ~ 380 pb, and DNA quantification was confirmed by Qubit 4 fluorometer (Thermo Fisher Scientific). Next, a double purification with magnetic beads was carried out (AMPure XP, Beckman Coulter, Brea, California, USA). The last quality control at this point was evaluated using HS Bioanalyser (Agilent Technologies, Santa Clara, California, USA) to assess the size of the library and check that the primers had been removed. DNA sequencing was conducted using Illumina MiSeq™ System (Illumina, San Diego, California, USA).

### Data processing and decontamination analysis

Raw sequencing data were imported in a PairedEndFastqManifestPhred33 input format to Quantitative Insights Into Microbial Ecology version 2 (QIIME2) software (v.2023.8). The quality control analysis was conducted applying the *denoise* function with DADA2 algorithm, and the reads’ quality scores were visualised using Fastqc software [49]. Low-quality regions were trimmed considering a quality score below 25 to create high quality forward and reverse reads. The good quality sequences were classified in amplicon variant sequences (ASVs) and the taxonomic assignment at the genus level was performed against the SILVA v138.1 database. Finally, rarefaction curves were generated to evaluate the sequencing depth, allowing the exclusion of samples in which the sequencing process did not adequately capture microbial diversity.

Due to the low bacterial biomass in endometrial and urine samples, in silico decontamination procedure was conducted using microDecon R package, which corrects for potential contaminants based on taxa detected in negative controls, thereby improving the accuracy of results from low-biomass microbiomes [50].

### Statistical analysis

Statistical analysis and data visualisation were conducted in R software (v.4.4.1.) under RStudio (v.4.4.1.), using phyloseq [51], vegan [52], microViz [53], ggplot2 [54] and ggpicrust2 packages [55]. Within-sample diversity (i.e., alpha-diversity) was estimated by Shannon diversity index and observed genera (Richness) using the *diversity* and *specnumber* functions of the vegan R package. Analysis of Covariance test (ANCOVA) was conducted considering the age, body mass index (BMI), diagnosis, the use of antimicrobials (antibiotics, antivirals and antifungals) and the participant identifier as covariates for statistical comparisons between different body sites. Between-sample dissimilarity (beta-diversity) was visualised using Principal Coordinates Analysis (PCoA) and significance testing performed by Permutational Analysis of Variance (PERMANOVA) using *adonis* function from vegan R package. Additionally, pairwise PERMANOVA using *pairwiseAdonis* function was conducted to evaluate dissimilarities between pairs of study groups. The differential abundance of bacterial genera between body sites was evaluated using the metagenomeSeq R package [56]. This package allowed the normalisation of the data considering their paired character. The log Fold Change (LogFC) and the adj *p*-value were obtained for several comparisons. All *p*-value were corrected using Benjamini-Hochberg false discovery rate (FDR) [57]. *P*-values  < 0.05 implied a statistically significant differential abundance of a bacterial genus between the study groups. To identify microbial associations within the female reproductive tract, Spearman’s correlations between microbes across body sites were performed and visualised in heatmaps using corrplot R package [58]. An intra-individual microbial profile of the female reproductive tract was established for each participant using the Bray-Curtis distance to compare the similarity between microbiomes. A sensitivity analysis was performed by repeating the main analyses exclusively within the male factor infertility subgroup (*n* = 28); all participants with underlying gynaecological pathologies were excluded from this sub-analysis to verify whether the observed microbial patterns remained consistent with those of the entire study population.

Diversity and differential abundance analyses were also performed to evaluate the differences between endometriosis, RIF and control (women from couples diagnosed with male factor infertility) groups, as these represented the largest cohorts in our study. Our primary differential abundance testing was executed using metagenomeSeq, which utilised zero-inflated models to handle sparse data while controlling for age, BMI and the use of antimicrobials as covariates. Additionally, as a complementary exploratory approach to visualise community-wide differential taxonomic abundance, a nonparametric Wilcoxon rank-sum tests [59] with FDR correction was carried out. Differential taxonomic abundance in each location was represented by a differential heat tree using metacoder R package [60]. Finally, the software PICRUSt2 (v.2.6.1) (Phylogenetic Investigation of Communities by Reconstruction of Unobserved States) was employed to predict the functional profiles of the microbiome based on Kyoto Encyclopedia of Genes and Genomes (KEGG) database and Enzyme Commission (EC) annotations. Comparative analysis of KEGG pathway and EC annotations were performed using ALDEx2 R package. Principal Component Analysis (PCA) and heatmaps were used to visualise and predict metabolic pathways, inferring the functional profile of the microbiome and the relative abundance of identified pathways.

## Results

### Study population

The study population consisted of 136 women prior to undergoing infertility treatment with Assisted Reproduction Techniques (ARTs); however, due to incomplete sample set (each participant needed to provide samples from 5 different sites), the final study population was comprised of 104 women (Fig. 1). The average age and BMI of the participants were 34 years and 23.36 kg/m^2^, respectively. The causes of infertility included the following conditions: adenomyosis (*n* = 1), endometriosis (*n* = 24), endometritis (*n* = 4), male factor infertility (*n* = 28), PCOS (*n* = 3), RIF (*n* = 22), RPL (*n* = 8), tubal factor (*n* = 3), unexplained infertility (*n* = 7), and uterine fibroid (*n* = 4) (Table 1). Seventy-four patients were included in the condition-based analysis: endometriosis (*n* = 24), RIF (*n* = 22), and male factor infertility as controls (*n* = 28). Patient characteristics are summarised in Table 2.

**Table 1:** Demographic and clinical characteristics of the patients included in the study

Study population (N = 104)	
Age (years)	34 ± 3.80
Weight (kg)	63 ± 12.21
Height (m)	1.65 ± 0.06
BMI (kg/m^2^)	23.36 ± 4.11
Diagnosis n (%)	
Adenomyosis	1 (0.96)
Endometriosis	24 (23.08)
Endometritis	4 (3.85)
Male factor	28 (26.92)
PCOS	3 (2.88)
RIF	22 (21.15)
RPL	8 (7.69)
Tubal factor	3 (2.88)
Unexplained	7 (6.73)
Uterine fibroid	4 (3.85)

Data presented as mean ± standard deviation or frequency, as appropriate. BMI (body mass index); PCOS (polycystic ovary syndrome); RIF (recurrent implantation failure); RPL (recurrent pregnancy loss)

**Table 2:** Clinical characteristics of the patients included in the condition-based analysis

Study population (N = 74)				
Patient features	Endometriosis (n = 24)	RIF(n = 22)	Control(n = 28)	P-value
Age (years)	33.33 ± 3.42	34.91 ± 3.78	34.93 ± 3.88	> 0.05
BMI (kg/m^2^)	23.42 ± 3.85	24.43 ± 4.06	24.56 ± 3.90	> 0.05
Antimicrobials (n/N)	3/24	5/22	8/28	> 0.05

Data presented as mean ± standard deviation. T-student and chi-square tests were performed. T-test for age revealed a *p*-value of 0.16 between endometriosis and RIF groups, 0.10 between endometriosis and male factor groups and 0.75 for RIF and male factor groups. For the BMI the following *p*-values were obtained for the comparisons: 0.94 (endometriosis vs. RIF), 0.87 (endometriosis vs. male factor) and 0.90 (RIF vs. male factor). The chi-square test was used to evaluate the antimicrobials taken; endometriosis, RIF and male factor groups revealed a P-value of 0.29, 0.96 and 0.35, respectively. BMI (body mass index); RIF (recurrent implantation failure)

### Data processing

After taxonomic assignment at the genus level in each sample type, we identified: 177 bacterial genera in the vaginal samples, 399 in the cervical samples, 1058 in the endometrial samples, 958 in the urine samples and 518 in the rectal samples (Supplementary Tables S1). Due to the low-biomass nature of the endometrial and urine samples, a decontamination correction was performed. As a result, 319 (30.15%) and 229 (23.90%) bacteria were identified as contaminants and were subsequently removed from their respective datasets (see Supplementary Figure S1). The average number of reads (mean ± SD (standard deviation)) in each site was 59,650 ± 30,542.13 in the vaginal samples, 57,472 ± 72,655.89 in the cervical samples, 38,736.50 ± 31,653.14 in the endometrial samples, 41,782 ± 26,781.39 in the urine samples, and 52,726.50 ± 17,975.44 in the rectal samples.

### Characterisation of the microbial profiles

**Fig. 2:**
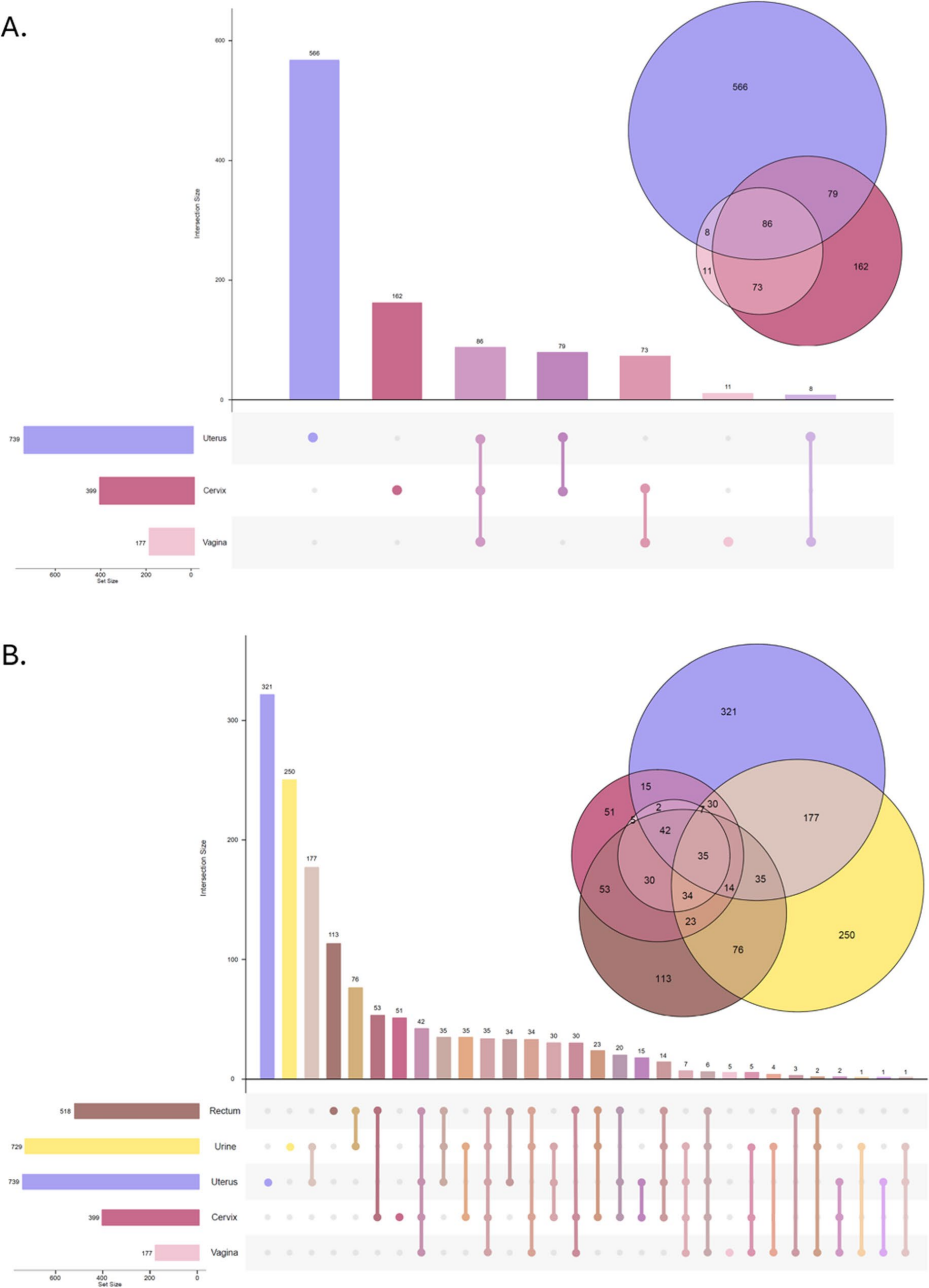
UpSet plot and Venn Diagram showing total bacterial genera identified in the study population. The size of the circle is proportional to the number of bacteria. (**A**) Bacteria shared within the female reproductive tract (vagina, cervix and uterus). (**B**) Bacteria shared between the female reproductive tract (vagina, cervix and uterus) and adjacent sites (urine and rectum)

After data processing, we identified 177 bacterial genera in the vaginal samples, 399 in the cervical samples, 739 in the endometrial samples, 729 in the urine samples and 518 in the rectal samples. When comparing microbial profiles between the different body sites, we identified 159 bacterial genera shared between the vagina and cervix, 94 between the vagina and uterus, and 165 between the cervix and uterus, while 86 bacteria were present throughout the female reproductive tract (i.e., vagina, cervix and uterus) (Fig. 2A). In the urine samples, 89 bacterial genera were shared with the vagina, 181 with the cervix, 305 with the uterus, and 224 with the rectum. In rectal samples, 151 genera were shared with the vagina, 250 with the cervix, and 185 with the uterus (Fig. 2B).

**Fig. 3:**
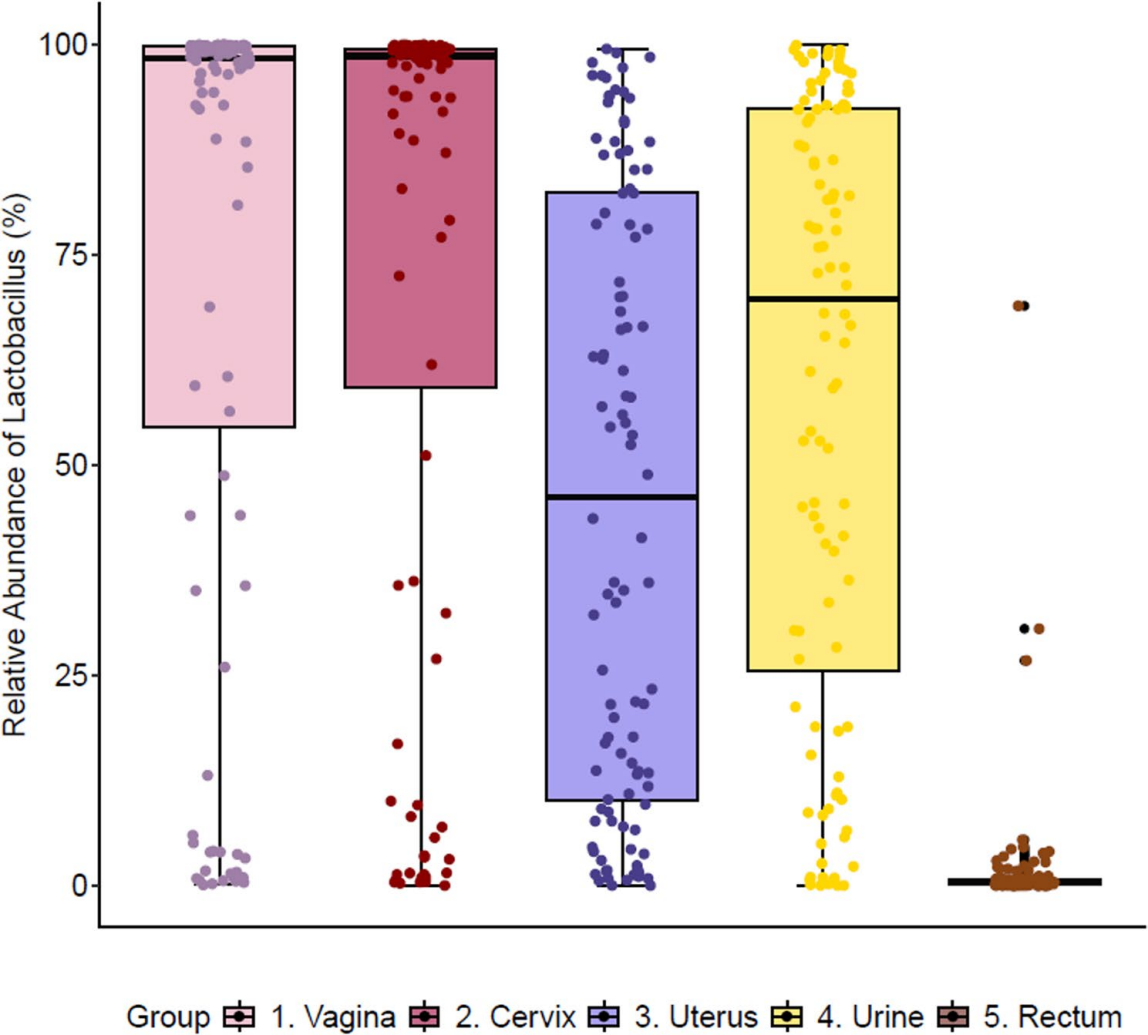
Dot-boxplot of the relative abundance of *Lactobacillus* in each body site. Each dot represents the relative abundance of *Lactobacillus* for each participant. The bold line within the box shows the median of each group

The microbial composition along the reproductive tract as well as in the urine showed a dominance of *Lactobacillus* (Fig. 3). Specifically, the relative abundance of *Lactobacillus* was 98.42% [55.48; 99.75] (median [quartil1; quartil3] in the vaginal samples, 98.65% [59.25; 99.51] in the cervical samples, 46.26% [10.07; 82.34] in the endometrial samples and 69.73% [25.49; 93.36] in the urine samples. In contrast, in the rectal samples the abundance of *Lactobacillus* decreased to 0.42% [0.08; 0.72].

**Fig. 4:**
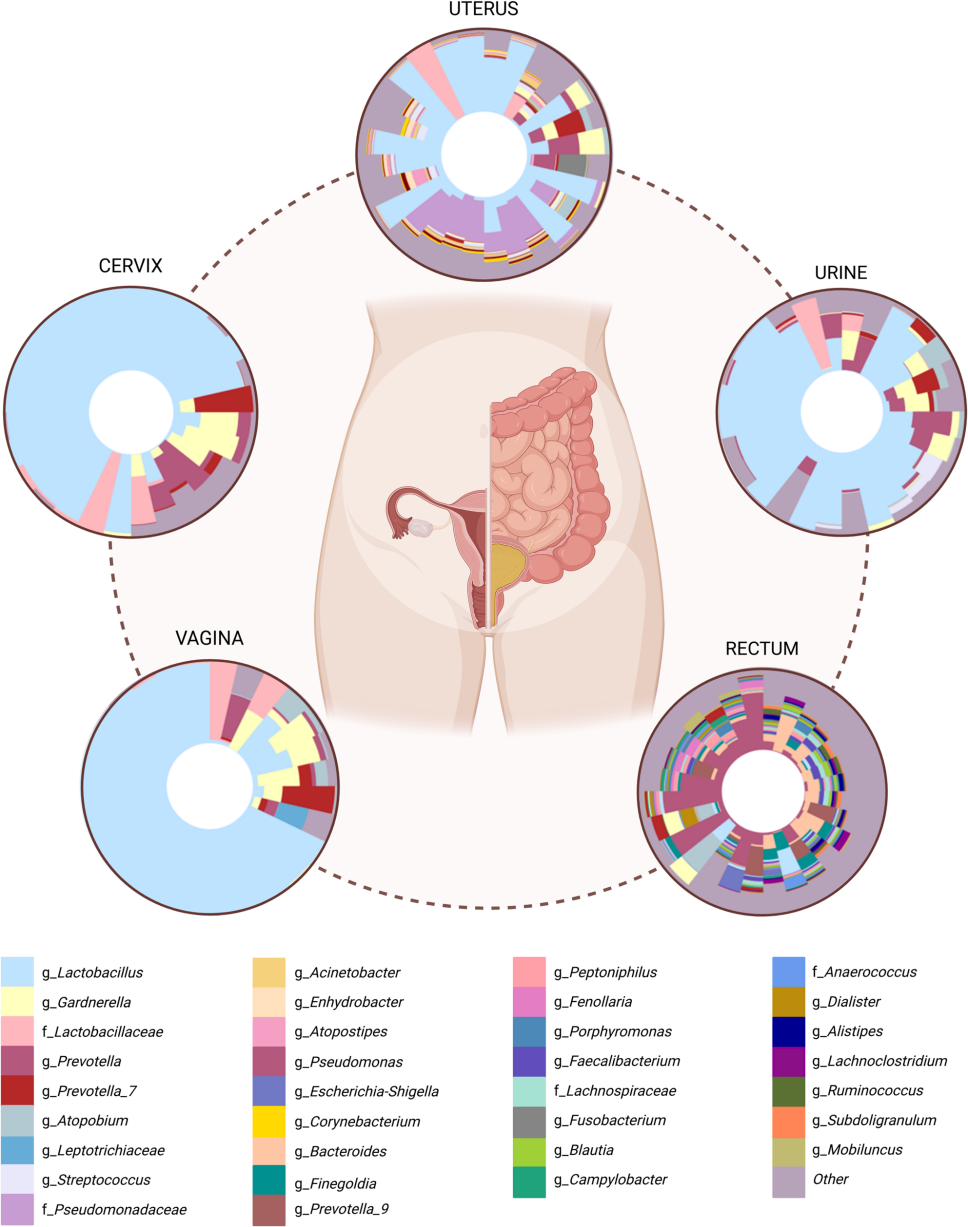
Microbial composition across the analysed body sites. Iris plots represent those bacterial genera with a relative abundance > 1%. The bacterial genera whose relative abundance was < 1% were grouped together and labelled as “Other”

The most abundant genera in the vagina and the cervix were entirely shared, while those between the lower reproductive tract and urine samples were partially shared, including *Gardnerella*,* Prevotella*, *Prevotella_7*, *Streptococcus* and a member of Lactobacillaceae family (Fig. 4). The endometrial samples had a more diverse composition than the lower reproductive tract. The rectal samples showed the highest microbial diversity, where *Prevotella* was the most abundant genus (relative abundance = 10.94 [3.54; 21.85]), followed by *Bacteroides* (relative abundance = 4.46 [1.78; 9.98]), *Finegoldia* (relative abundance = 2.61 [0.81; 6.01]) and *Peptoniphilus* (relative abundance = 2.36 [1.00; 4.49]) (Fig. 4).

### Differential microbial profiles across the body sites

**Fig. 5:**
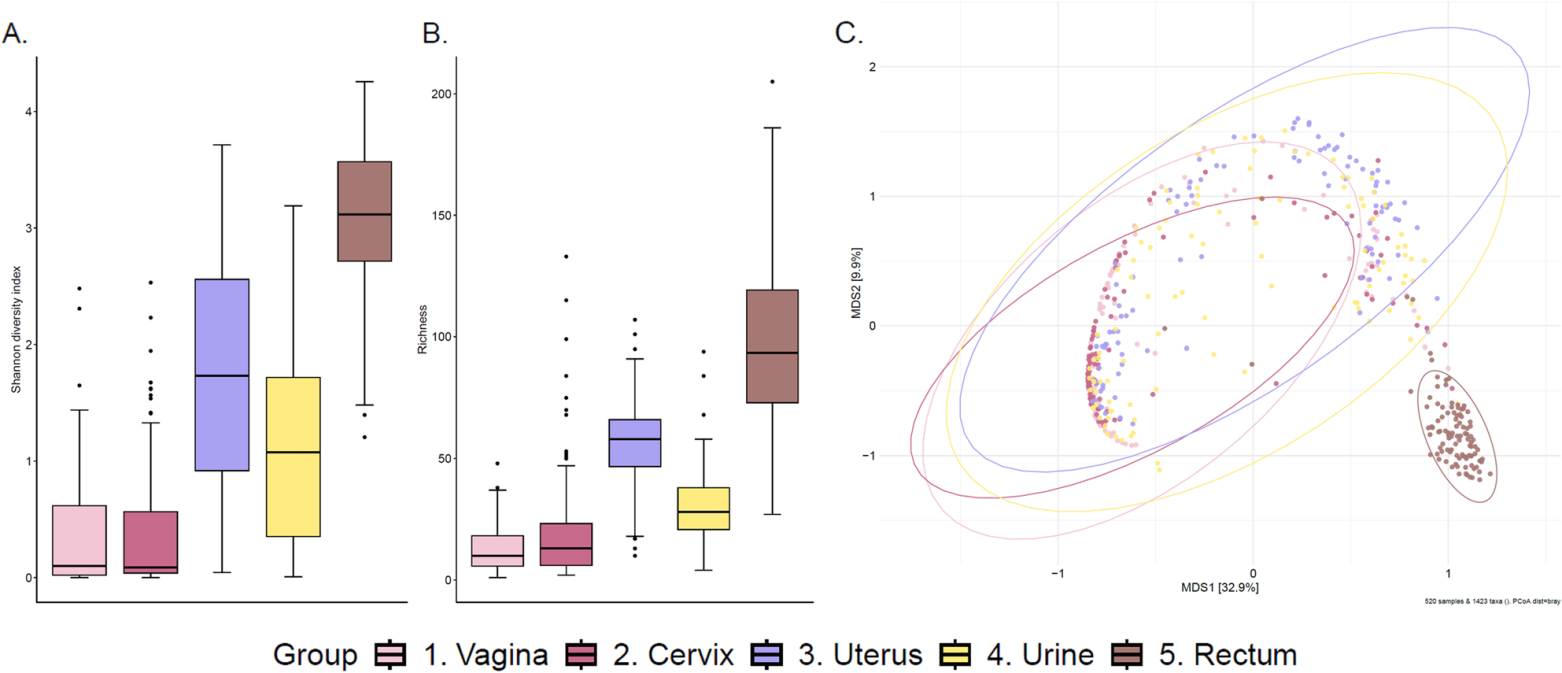
(**A**, **B**) Alpha-diversity evaluated by Shannon diversity index and Richness, respectively. (**C**) Beta-diversity represented using a principal coordinate analysis (PCoA) based on the Bray-Curtis distance

Microbiome diversity and differential abundance analyses were performed to evaluate the potential microbial differences between the body sites. Significant differences in alpha-diversity analysis were detected among the body sites using ANCOVA test for both Shannon diversity index and Richness (both adj *p*-value = 2E-16). Notably, the participant identifier covariate was also significant in both models (adj *p*-value = 8.61E-05 for the Shannon diversity index and adj *p*-value = 0.001 for Richness), meaning that part of the variance in alpha-diversity is explained by the study design, as each participant contributed one sample from each anatomical site analysed. Vaginal and cervical samples presented the lowest microbial diversity, with no significant differences in Shannon diversity index between these locations, while uterus and urine showed a higher alpha-diversity (Fig. 5A and B). Rectal samples obtained the highest score in alpha-diversity, which indicates a high microbial variance within-sample (Fig. 5A and B). The PCoA plot showed that the vaginal, cervical, endometrial, and urine samples clustered closely, suggesting that these body sites share similar microbial compositions, while rectal samples displayed a different distribution indicating a distinct microbial community structure (Fig. 5C). Beta-diversity analysis confirmed a significant overall dissimilarity across the five anatomical sites evaluated (Global PERMANOVA test, adj *p*-value = 0.001, R^2^ = 0.259). In addition, several covariates significantly contributed to the observed variance: age (adj *p*-value = 0.011, R^2^ = 0.003), antimicrobials use (adj *p*-value = 0.038, R^2^ = 0.002), gynaecological diagnosis (adj *p*-value = 0.001, R^2^ = 0.032) and, most importantly, the participant identifier (adj *p*-value = 0.001, R^2^ = 0.280), meaning that covariates reflect both biological influences and design-related factors. Then, pairwise comparisons were conducted, and the only non-significant result was observed between the vaginal and cervical microbial communities (Pairwise PERMANOVA test, adj *p*-value = 0.052, R^2^ = 0.002) (See Supplementary Table S2).

For differential abundance analysis, a threshold of 0.01% relative abundance was applied to remove those bacterial genera that were under-represented. Therefore, from the 1,423 total genera, we identified 220 bacteria which were considered in the subsequent analysis. Differential abundance analyses were performed between the body sites. Along the female reproductive tract, 15 bacteria were differentially abundant between the vagina and cervix, 166 between the vagina and uterus and 172 between the cervix and uterus (all adj *p*-values < 0.05, Supplementary Tables S3). Additionally, we detected 74, 111, 169 and 168 differentially abundant bacteria when compared the urine to the vagina, cervix, uterus and rectum, respectively (all adj *p*-values < 0.05, Supplementary Tables S3). Further, 171, 182 and 168 bacteria were differentially abundant when comparing the rectum to the vagina, cervix and uterus, respectively (all adj *p*-values < 0.05, Supplementary Tables S3).

### Microbial interactions in the female reproductive tract

**Fig. 6:**
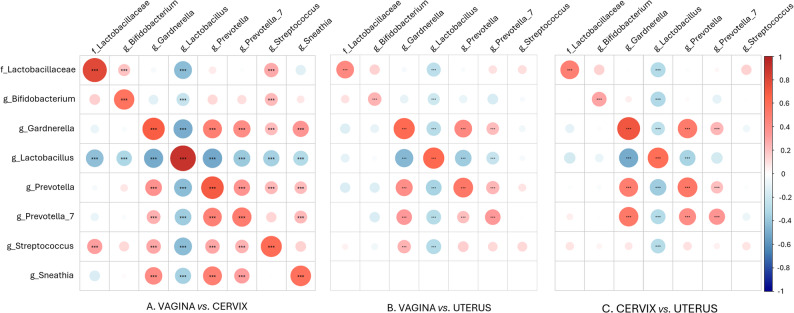
Correlation analysis of microbial abundance between vagina, cervix and uterus. Heatmaps represent those common bacterial genera between sites with a relative abundance > 1%. Associations were performed based on Spearman’s correlation. Positive correlations are displayed in red and negative correlations in blue. Colour intensity and size of the circles are proportional to the correlation coefficients. Correlation results were considered statistically significant according to the following *p*-values: * *p*-value < 0.05, ** *p*-value < 0.01 and *** *p*-value < 0.001

To further explore microbial interactions, correlation analyses were performed using a 1% relative abundance threshold, assessing co-occurrence patterns of bacterial genera with > 1% relative abundance within the female reproductive tract. Heatmaps showed that *Lactobacillus* were strongly negatively correlated with the rest of the genera evaluated between the vagina, cervix and uterus. By contrast, other genera as *Gardnerella*,* Prevotella* or *Prevotella_7* showed positive correlations among them (Fig. 6).

**Fig. 7:**
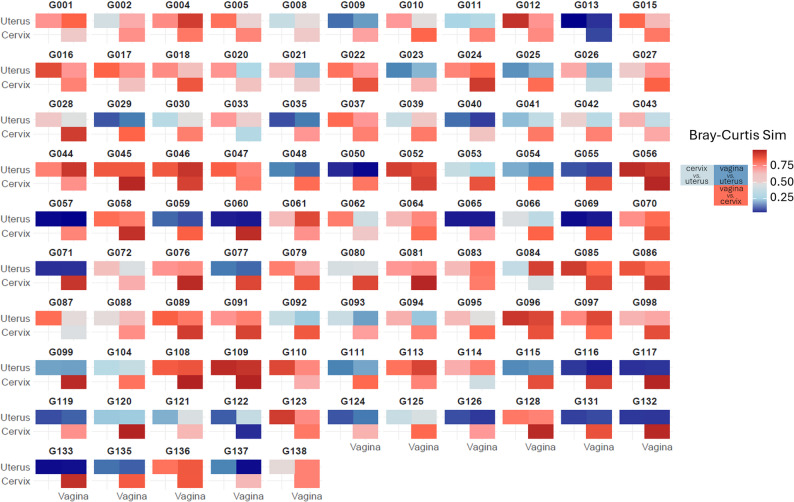
Intra-individual microbial profiles in the female reproductive tract. Heatmaps showing microbial comparisons between vagina, cervix and uterus for each patient based on Bray-Curtis distance. Greater similarity percentages are displayed in red and lesser in blue. Colour intensity is proportional to the level of similarity

An intra-individual profile was generated for each participant to compare microbial communities across reproductive tract sites (Fig. 7 and Supplementary Table S4). Bray-Curtis similarity was calculated, showing 78.93% [67.43; 78.93] (median [quartil1; quartil3] of concordance between vaginal and cervical samples, which reflects a strong microbial resemblance within the lower reproductive tract. Comparisons involving the endometrial microbiome showed lower similarity; 49.08% [21.50; 49.08] for the vagina–uterus and 54.69% [17.45; 54.69] for the cervix–uterus. At the individual level, most women (*n* = 97; 93.27%) showed high similarity (> 50%) between the vagina and cervix. Within this group, 43 individuals had high similarity for both the vagina–uterus and the cervix–uterus, while 42 women had low similarity (< 50%) for both. Two had high similarity for the vagina–uterus but low for the cervix–uterus, and 10 women showed the reverse. A smaller subset (*n* = 7; 6.73%) presented low similarity between the vagina and cervix. Taken together, these patterns indicate that, although variation exists in intra-individual similarity across the reproductive tract, high concordance between the vaginal and cervical microbial profiles was the dominant trend.

### Sensitivity analysis in control women

Given the clinical heterogeneity of the overall cohort and the presence of underlying gynaecological conditions, a sensitivity analysis including 28 women with male factor infertility diagnosis as controls was performed. The main findings for microbiome diversity, differential abundance and microbial correlations analyses, although attenuated, remained the same compared to those obtained for the whole study population (Supplementary Figures S2-7 and Supplementary Table S5).

### Microbial profiles in endometriosis and RIF

Alpha-diversity and beta-diversity analyses were carried out to evaluate the differences between endometriosis (*n* = 24), RIF (*n* = 22) and male factor (control) (*n* = 28) groups for each site. Alpha-diversity analysis showed that microbial diversity was similar between the women with gynaecological conditions such as endometriosis and RIF, and control women. But the diversity in the vagina (Shannon diversity index; *p*-value = 0.046 and Richness; *p*-value = 0.001) and in the cervix (Richness; *p*-value = 0.028) in women with endometriosis was higher than the women with RIF; however, in the rectum the diversity was higher in women with RIF (Shannon diversity index; *p*-value = 0.033) (Supplementary Figure S8A, S8B). Beta-diversity analysis revealed no significant differences between the groups in the vaginal, cervical and uterine samples. In the endometrium, it is important to note that the slight intergroup differences were influenced by the BMI covariate (Global PERMANOVA test, adj *p*-value = 0.028, R^2^ = 0.352). In the anatomically adjacent sites (urine and rectum) the diversity analyses did not reveal any statistically significant differences (Supplementary Figure S8C).

**Fig. 8:**
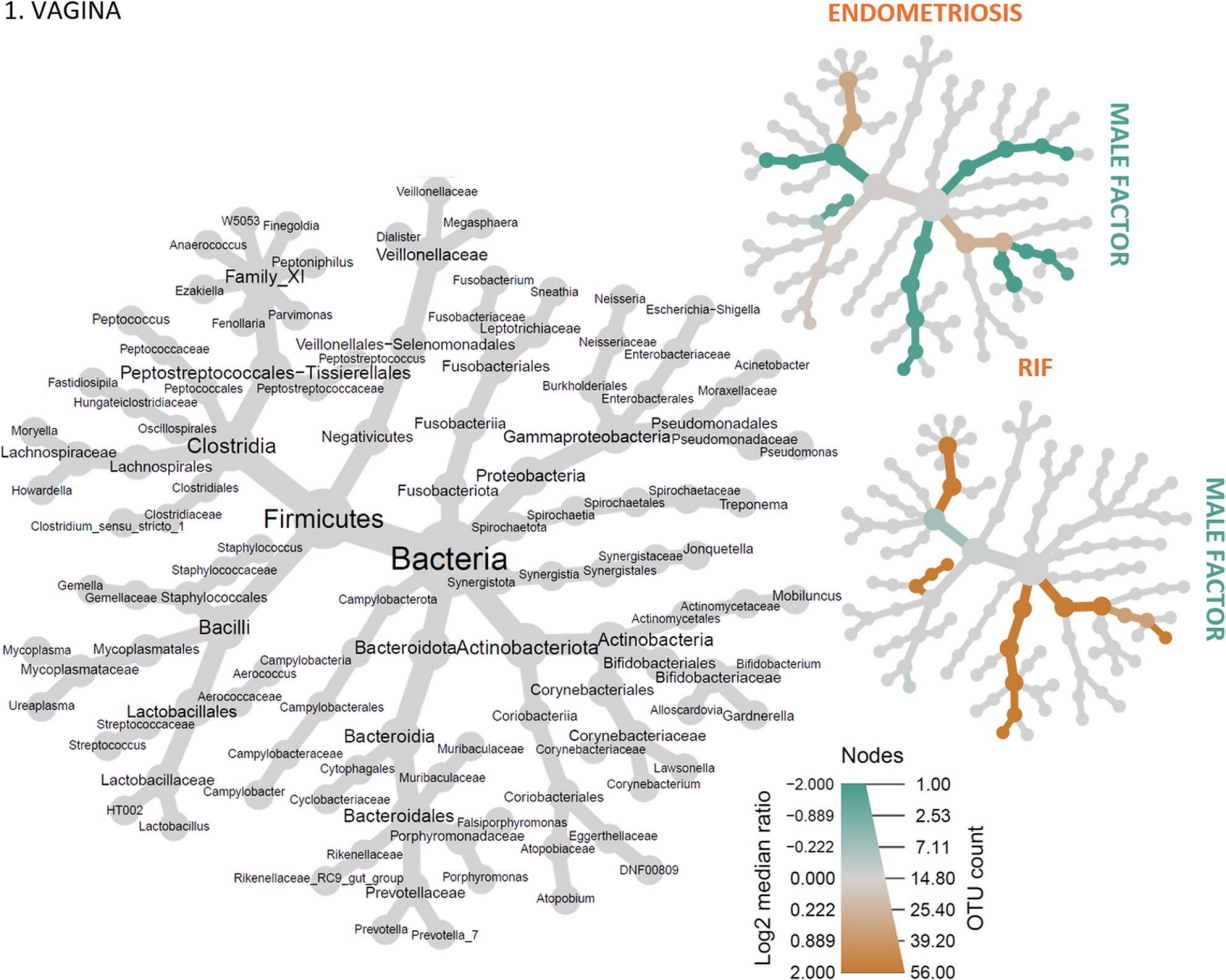
Heat tree of differential vaginal taxonomic abundance between the endometriosis and RIF groups versus the male factor group

For the primary differential abundance analysis, a relative abundance threshold of 0.01% was applied using metagenomeSeq R package, using zero-inflated models to rigorously account for sparse data and clinical covariates. In the vaginal samples, we identified 15 taxa with significant differences between endometriosis and controls, and 7 taxa between RIF and controls (Supplementary Table S6; non-adj *p*-value < 0.05); in the cervix, 5 taxa differed significantly in endometriosis vs. controls, and 4 taxa in RIF vs. controls (Supplementary Table S7; non-adj *p*-value < 0.05); in the endometrial samples, 9 taxa were significantly different in endometriosis vs. controls, and 13 taxa in RIF vs. controls (Supplementary Table S8; non-adj *p*-value < 0.05); in urine samples 19 taxa differed significantly in endometriosis vs. controls, and 17 taxa in RIF vs. controls (Supplementary Table S9; non-adj *p*-value < 0.05); and in the rectal samples, we identified 8 taxa in endometriosis vs. controls, and 12 taxa in RIF vs. controls (Supplementary Table S10; non-adj *p*-value < 0.05). None of these taxa remained statistically significant after the FDR correction, except for the four genera identified in the vaginal samples. An unidentified genus from Veillonaellaceae family (logFC = -2.17; adj *p*-value = 0.04) was more abundant in endometriosis group, while *Acinetobacter* (logFC = 2.24; adj *p*-value = 0.02), *Bifidobacterium* (logFC = 3.75; adj *p*-value = 0.03) and *Howardella* (logFC = 2.01; adj *p*-value = 0.03) genera were more abundant in the control group. To complement this with a community-wide visual overview, differential heat trees were constructed using non-parametric Wilcoxon rank-sum tests to screen for identified shifting trends between the groups. For each location analysed, two differential heat trees were represented to compare the endometriosis and RIF groups with the control group, where a filter of 0.01% of relative abundance was applied (Fig. 8, Supplementary Figures S9-12). The genera with un-corrected *p-*values < 0.05 are represented in Table 3 and Supplementary Table S11-S12. No genus remained statistically significant after the FDR correction at each location.

**Table 3:** Differential heat-tree genera between pathology and control groups in each studied location evaluated using a nonparametric Wilcoxon rank-sum tests ( *p*-value < 0.05; adj *p*-value > 0.05)

	Endometriosis vs. Controls	RIF vs. Controls
CERVIX	*Streptococcus*	*Actinomyces*
		***Gardnerella***
UTERUS	***Fusobacterium***	*Porphyromonas*
		***Gardnerella***
		***Alloprevotella***
		*Brevibacterium*
		***Atopobium***
URINE	***Streptococcus***	
	***Finegoldia***	
RECTUM	***Peptoniphilus***	***Bilophila***
	*Staphylococcus*	
	***Lawsonella***	
	***Finegoldia***	
	***Varibaculum***	
	***Campylobacter***	
	***Anaerococcus***	

Genera in bold are more abundant in the pathology group. In the vagina none of the bacterial genera presented differential abundances between the studied groups. RIF (recurrent implantation failure)

Functional prediction analysis using the software PICRUSt2 was performed in order to identify the possible metabolic pathways involved in endometriosis and RIF. Two databases (KEGG and EC) were used in the functional predictions from the 16 S rRNA gene data; however, no differential pathways were identified between the groups (Fig. 9, Supplementary Figures S13-21).

**Fig. 9:**
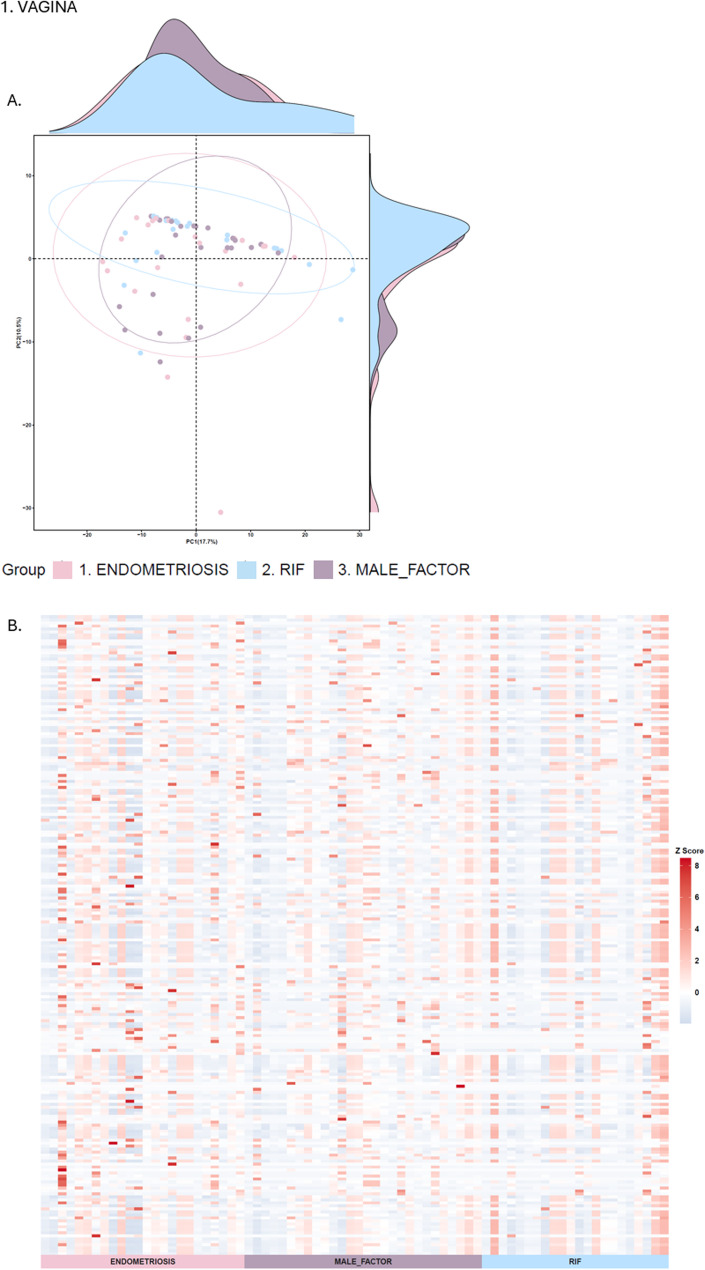
Prediction of functional profiles based on KEGG database in vaginal samples. (**A**) Principal Coordinate Analysis (PCA) of predicted metabolic pathways using PICRUSt2. The plot shows the distribution of endometriosis (pink), RIF (blue) and male factor (grey) vaginal samples in the space of the first two principal components (PC1 and PC2). The correlation between the original variables and the principal components was evaluated using the Pearson correlation coefficient. (**B**) Heatmap of significantly different metabolic pathways inferred by PICRUSt2 between groups in vagina. Each row represents a metabolic pathway and each column an individual sample. Z-score values, shown by the colour scale, were calculated by normalizing pathway abundances to highlight significant differences. Hierarchical clustering was performed using Euclidean distance and average linkage method. RIF (recurrent implantation failure)

## Discussion

### Microbial communities in the female reproductive tract and adjacent sites

The female lower reproductive tract harbours a complex community of microorganisms, including aerobic and facultative anaerobic species. Similar taxa have also been detected in the endometrium, however, whether the uterus hosts a distinct resident microbiome and whether there is a common microbial composition throughout the urogenital tract remains under debate.

To address this question, we characterised the microbiome across a continuum of anatomical locations comprising the urogenital-gut axis: urine, vagina, cervical canal, uterine cavity and rectal mucosa. This anatomical gradient was selected to enable direct comparison of microbial communities across neighbouring but functionally distinct niches. Our results revealed a continuous bacterial transition from the lower reproductive tract (vagina and cervix) to the uterus, alongside marked differences with the rectal microbiome. Along this ascending path, there is an increase in the richness and diversity of bacterial genera, in contrast to a decrease in the bacterial biomass. Moreover, the vaginal and cervical microbiomes present a high similarity with only a few bacterial genera being differentially abundant. Our study results highlight the *Lactobacillus-*dominance > 98% and the presence of some genera as *Gardnerella*, an unidentified genus of Lactobacillaceae family, *Bifidobacterium*, *Streptococcus*, *Prevotella* and *Prevotella_7* in both lower reproductive tract sites. Furthermore, the intra-individual microbial profile of each woman reveals an individual microbial fingerprint, with vaginal and cervical microbiomes sharing approximately 80% similarity and exhibiting substantial niche overlap. However, the endometrial microbiome differs from the lower reproductive tract, showing around 50% of similarity with these sites. These findings suggest a bacterial transition from the vagina to the cervical site that could be facilitated by the absence of an anatomical barrier as proposed in previous studies [17]. However, the cervical canal and the cervical mucus act as a semi-permeable barrier and a selective antimicrobial medium, respectively, thereby restricting the full microbial transfer from the vagina-cervix to the uterus [18, 61]. Despite this fact, the endometrial microbiome may receive an important influence from the lower reproductive tract, at least on detectable DNA level. The detection of *Lactobacillus* in the endometrium represented around 46% in our study, being the most abundant genus in this site. Besides, several bacterial genera with the highest abundance in the uterus such as *Gardnerella*,* Prevotella*,* Prevotella_7* and an unidentified genus of Lactobacillaceae family were shared with the vagina and cervix. In summary, our study reflects not only an intra-individual microbial variability within the system that is attenuated in the lower reproductive tract sites, but also the presence of an individual core microbiome of each studied location.

In this context, the microbial interactions throughout the female reproductive tract are represented by a constant bacterial competition to colonise each niche. Our data indicate that *Lactobacillus* may maintain the homeostasis by establishing a negative relationship with the non-dominant bacteria along the path, whereas other bacterial genera (e.g., *Gardnerella*,* Prevotella* or *Atopobium*) show neutral or cooperative interactions between them. This reflects how *Lactobacillus-*dominance, specifically in the lower reproductive tract is able to modify its surroundings through lactic acid production and to inhibit the growth of pathogenic bacteria, which could alter the microbial homeostasis and reproductive function through this non-closed system [8, 11, 12, 62–64].

Taking this concept a step further, previous research has recognised that the female reproductive system is not an isolated entity, but forms part of a network of interconnected body sites [30, 42, 65]. Different pathways involving the urinary and intestinal tracts have been described, establishing the vagina-bladder and vagina-gut axes [29, 30, 65]. Two main mechanisms underlie these bidirectional interactions: (i) the direct translocation of microorganisms facilitated by anatomical proximity, and (ii) the indirect systemic communication mediated by metabolites, hormones, and the immune system [29, 30]. The vagina-bladder axis is supported by the significant bacterial similarity found in both locations, suggesting the genitourinary microbiome may be a reliable indicator of the vaginal microbial state [66]. Furthermore, the rectum acts as a microbial reservoir that directly influences the composition of the vaginal and urinary microbiomes [29, 67, 68]. This evidence indicates that microbial imbalance in any of these sites could be a factor that alters reproductive health. Therefore, our study contributes a new perspective to this field by including the microbial analysis of the urine and rectum.

Our study results highlight a shared microbial composition in the reproductive tract with the urinary microbiome dominated by *Lactobacillus (*~ 70%) genus followed by other genera such as *Prevotella*,* Gardnerella*,* Streptococcus*, *Escherichi-Shigella*, an unidentified genus of Lactobacillaceae family, *Prevotella_7*,* Atopobium* and *Peptoniphilus*. The high similarity could be influenced by first sampling the mid-catch urine and subsequently sampling reproductive tract. Nevertheless, these urogenital microbial similarities are consistent with previous studies [35, 66]. It has been suggested that the vaginal microbiome acts as a reservoir of uropathogens triggering dysbiosis in the urobiome which is related to inflammatory process and inflammatory urinary diseases [29, 69]. This bidirectional pathway corroborates that urine may represent a clinically relevant microbial ecosystem, influencing female urogenital health. We also analysed the rectal mucosa which represents the anatomical site most closely positioned to the posterior part of the reproductive tract. Despite this proximal location, the rectal microbiome was markedly different from that of the urogenital tract, showing the greatest bacterial diversity and the lowest abundance of *Lactobacillus*. This distinction suggests that direct microbial translocation due to anatomical proximity is less likely to be the primary mechanism shaping the urogenital tract, pointing instead toward systemic pathways (e.g. effector molecules, estrobolome) as the more plausible link between the gut and the female reproductive tract. Microbial activity within the gut modulates oestrogen metabolism via bacterial communities that are able to deconjugate inactive oestrogen back to its active form that can be liberated to the circulation [70]. The resulting metabolic processes may interact across the female reproductive tract affecting the distal vaginal epithelium and reaching the cervical epithelium and endometrial lining, thereby modulating local physiology and contributing to the development of conditions such as endometriosis, endometrial hyperplasia, and endometrial and ovarian cancer [42, 71].

### Microbial communities in endometriosis and RIF

Endometriosis and RIF are the two gynaecological pathologies in which the uterine factor is closely involved, but their underlying mechanisms remain unclear. In both conditions, the implication of the microbial communities in the reproductive tract have been studied and specific bacterial associations have been reported, but with inconclusive results across studies [72, 73]. To address these discrepancies, our study characterises the microbial composition in women with endometriosis, RIF, or control women through the urogenital-gut axis, providing more holistic microbiomes view than of a sole single site, being the focus of the previous studies. Also, our study is the first to characterise the urobiome in RIF, and the research on the rectal microbiome in RIF is preliminary, with a single study published to date [74]. Similarly, studies of the urobiome in endometriosis are lacking, with one study performed so far [75].

Our study results show no significant differences in the functional analyses and in either alpha- or beta-diversity analyses in any of the sites analysed, reflecting similar microbial community structures between women with endometriosis, RIF, or control women along the urogenital tract and rectal axis. In line, also our previous whole metagenome study on a cohort of 1000 women did not detect any microbial difference in the gut between women with and without endometriosis [70].

On the single bacterial community levels, however, we detected slight compositional alteration in the vaginal microenvironment where an enrichment of an unidentified genus of Veillonaceae family and a decrease of *Bifidobacterium*, *Acinetobacter* and *Howardella* were associated with endometriosis. In line with previously published findings, an increase of members of Veillonaceae family have been identified within the vaginal microbiome in endometriosis [76–78], and these taxa have been further linked to vaginal inflammation and dysbiosis [79]. In addition, this bacterial family and *Acinetobacter* genus have been associated with endometriosis in cervical and endometrial samples [19, 80]. Conversely, a reduction in abundance of *Acinetobacter spp.* have been reported in the endometrium of women with endometriosis [81]. This may suggest a potential bacterial interconnection along the reproductive tract, facilitated by the uterine peristaltic contractions that are more frequent in endometriosis [82, 83]. Further, the decline of *Bifidobacterium* in the vaginal microenvironment of women with endometriosis has been detected also previously, supporting further our findings [77, 84]. However, a bidirectional Mendelian randomization study of the gut microbiota has suggested a protective role for *Bifidobacterium* against endometriosis and has indicated that *Howardella* abundance may be influenced by the presence of the disease [85]. Although these findings refer to the gut microbiota, the systemic communication [29, 30] linked with the anatomical factors such as a short anogenital distance may promote bacterial transfer from the rectal microbial reservoir to the vagina, including these two gut bacteria implicated in the oestrogen metabolism [85–87]. This is especially relevant given the oestrogen-dependent nature of endometriosis, as the estrobolome plays important role in the development and progression of this disease. These microorganisms with β-glucuronidase activity can deconjugate inactive oestrogens back into the active form, modulating thereby the hormone circulating levels [42, 44].

Interestingly, our study detected a potential overabundance, although not statistically significant level after multiple testing correction, of *Fusobacterium* in the endometrium in women with endometriosis, corroborating its involvement in endometriosis pathogenesis, which was previously demonstrated [88]. Nevertheless, a recent study in endometrial samples reported no association between *Fusobacterium* and endometriosis [89]. The inconsistency in findings could be attributed, at least to some extent, to the differences in disease stages, phase of the menstrual cycle, stage of reproductive life, patient phenotype, tissue type, and sampling and analysis methods.

### Limitations and strengths

Although providing new information, our study has some limitations that should be highlighted. By evaluating the microbiome using 16S rRNA gene sequencing technique, we can reliably identify bacteria at the genus level [90, 91]. Also, the analysis of V4 region only, could have limited identification of some bacterial genera, and could hinder detailed comparisons with previous studies, where other hypervariable regions of the 16S rRNA gene have been studied. Future studies aiming to identify bacterial species and functional microbes with RNA sequencing are warranted, although the whole metagenome sequencing analysis on low microbial biomass sites such as the endometrium and urine can be methodologically challenging [92]. Also, endometrial sampling transcervically, even when using special double-shielded device (e.g., Tao Brush), cannot totally avoid microbial contamination from the lower reproductive tract, which may increase ‘artificially’ the detection of lactobacilli in the uterus [28]. Similarly, despite mid-catch protocols, contamination from the urethra and vulvovaginal region remains a challenge for urine collection.

Our study presents several strengths that support the results obtained. First, none of the women included in the study were undergoing hormonal treatment during the sample collection, and all exhibited natural menstrual cycles with sampling timed to the receptive phase endometrium. Cyclic fluctuation of microbial composition throughout the cycle has been shown [93], thus it is important to time study participants into the same cycle phase. Second, in our study population, paired samples were collected, i.e., all participants provided samples for all five anatomic sites analysed. Finally, the use of negative and positive controls throughout the study protocol together with the decontamination process used at low biomass sites improved the accuracy and reliability of the results.

## Conclusions

In conclusion, through the simultaneous characterisation of the female reproductive tract, urinary, and rectal microbiomes, our study offers a holistic perspective on the microbial landscape of the female urogenital-rectal axis, contributing to a broader understanding of its functional interconnections. Our results show shared microbiomes among the vagina, cervix and uterus, supporting the existence of a microbial continuum that interacts within this dynamic system, which also presents similarities with the urine microbiome. We found that the rectal microbiome has a significantly different composition to the other microbial niches analysed. Moreover, our findings show that although some intra-individual variation exists across the reproductive tract, inter-individual variability was considerably more pronounced, suggesting that individual factors may exert a stronger influence on microbial profiles than anatomical site. The microbial continuum of the urogenital-rectum axis did not associate with endometriosis or RIF in our study cohort, while microbiomes changes were detected, they did not reach the statistical significance after stringent decontamination and statistical corrections. Only in the vagina, single bacteria associated with endometriosis. Our study results suggest that the microbial differences on bacterial genus level might be modest and hard to detect, while a whole metagenome analysis, in the future studies, would provide more precise information of the potential microbes associating with reproductive health and disease.

## Supplementary Information

Supplementary Material 1: Supplementary Table S1. List of taxa identified at each of the study sites. Supplementary Table S2. Pairwise PERMANOVA results evaluating beta-diversity dissimilarities between groups, with covariates included in the model. Supplementary Table S3. Differential abundance analysis between body sites. Microbial taxa with a relative abundance > 0.01% were compared between groups using metagenomeSeq, which employs a zero-inflated Gaussian model to handle sparsity and variability in microbiome count data. Supplementary Table S4. Comparisons between vagina, cervix and uterus for each patient based on Bray-Curtis distance. Supplementary Table S5. Differential abundance analysis between body sites in sensitivity analysis. Microbial taxa with a relative abundance > 0.01% were compared between groups using metagenomeSeq, which employs a zero-inflated Gaussian model to handle sparsity and variability in microbiome count data. Supplementary Table S6. Differential abundance analysis between groups (endometriosis, RIF (recurrent implantation failure), and male factor) in vagina. Microbial taxa with a relative abundance > 0.01% were compared between groups using metagenomeSeq, which employs a zero-inflated Gaussian model to handle sparsity and variability in microbiome count data. Supplementary Table S7. Differential abundance analysis between groups (endometriosis, RIF (recurrent implantation failure), and male factor) in cervix. Microbial taxa with a relative abundance > 0.01% were compared between groups using metagenomeSeq, which employs a zero-inflated Gaussian model to handle sparsity and variability in microbiome count data. Supplementary Table S8. Differential abundance analysis between groups (endometriosis, RIF (recurrent implantation failure), and male factor) in uterus. Microbial taxa with a relative abundance > 0.01% were compared between groups using metagenomeSeq, which employs a zero-inflated Gaussian model to handle sparsity and variability in microbiome count data. Supplementary Table S9. Differential abundance analysis between groups (endometriosis, RIF (recurrent implantation failure), and male factor) in urine. Microbial taxa with a relative abundance > 0.01% were compared between groups using metagenomeSeq, which employs a zero-inflated Gaussian model to handle sparsity and variability in microbiome count data. Supplementary Table S10. Differential abundance analysis between groups (endometriosis, RIF (recurrent implantation failure), and male factor) in rectum. Microbial taxa with a relative abundance > 0.01% were compared between groups using metagenomeSeq, which employs a zero-inflated Gaussian model to handle sparsity and variability in microbiome count data. Supplementary Table S11. Differential abundance analysis of microbial taxa between endometriosis and control groups across all evaluated anatomical sites. Taxa with a relative abundance > 0.01% were analysed using the metacoder R package, which estimates effect sizes and statistical significance across taxonomic levels based on observed count differences and group contrasts. Supplementary Table S12. Differential abundance analysis of microbial taxa between RIF (recurrent implantation failure) and control groups across all evaluated anatomical sites. Taxa with a relative abundance > 0.01% were analysed using the metacoder R package, which estimates effect sizes and statistical significance across taxonomic levels based on observed count differences and group contrasts.

Supplementary Material 2: Supplementary Figure S1. (A) Percentages of true (purple) and contaminant (grey) reads in uterus samples by participant. (B) Percentages of true (yellow) and contaminant (grey) reads in urine samples by participant. Supplementary Figure S2. Venn Diagram showing total bacterial genera identified in sensitivity analysis population. The size of the circle is proportional to the number of bacteria. (A) Bacteria shared within the female reproductive tract (vagina, cervix and uterus). (B) Bacteria shared between the female reproductive tract (vagina, cervix and uterus) and adjacent sites (urine and rectum). Supplementary Figure S3. Dot-boxplot of the relative abundance of Lactobacillus in each body site. Each dot represents the relative abundance of Lactobacillus of each participant in sensitivity population. The bold line within the box shows the median for each group. Supplementary Figure S4. Microbial composition across the body sites in sensitivity population. Iris plots represent those bacterial genera with a relative abundance > 1%. The bacterial genera whose relative abundance were <1% were grouped together and labelled as “Other”. Supplementary Figure S5. Diversity analysis in sensitivity population (A, B) Alpha-diversity evaluated by Shannon diversity index and Richness, respectively. (C) Beta-diversity represented using a principal coordinate analysis (PCoA) based on the Bray-Curtis distance. Supplementary Figure S6. Correlation analysis of microbial abundance between vagina, cervix and uterus in sensitivity population. Heatmaps represent those common bacterial genera between sites with a relative abundance > 1%. Associations were performed based on Spearman’s correlation. Positive correlations are displayed in red and negative correlations in blue. Colour intensity and the size of the circles are proportional to the correlation coefficients. Correlation results were considered statistically significant according to the following p-values: * p-value < 0.05, ** p-value < 0.01 and *** p-value < 0.001. Supplementary Figure S7. Intra-individual microbial profile in the female reproductive tract (vagina, cervix and uterus) in sensitivity population. Heatmaps of microbial comparisons between vagina, cervix and uterus for each patient based on Bray-Curtis distance. Greater similarity percentages are displayed in red and lesser in blue. Colour intensity is proportional to the similarity level. Supplementary Figure S8. Diversity analysis between endometriosis (pink), RIF (recurrent implantation failure) (blue) and male factor (purple) groups. (A, B) Alpha-diversity evaluated by Shannon diversity index and Richness, respectively. (C) Beta-diversity represented using a principal coordinate analysis (PCoA) based on the Bray-Curtis distance. Supplementary Figure S9. Heat tree of differential cervical taxonomic abundance between the endometriosis and RIF (recurrent implantation failure) groups versus the male factor group. Supplementary Figure S10. Heat tree of differential endometrial taxonomic abundance between the endometriosis and RIF (recurrent implantation failure) groups versus the male factor group. Supplementary Figure S11. Heat tree of differential urine taxonomic abundance between the endometriosis and RIF (recurrent implantation failure) groups versus the male factor group. Supplementary Figure S12. Heat tree of differential rectal taxonomic abundance between the endometriosis and RIF (recurrent implantation failure) groups versus the male factor group. Supplementary Figure S13. Prediction of functional profiles based on KEGG database in cervical samples. (A) Principal Coordinate Analysis (PCA) of predicted metabolic pathways using PICRUSt2. The plot shows the distribution of endometriosis (pink), RIF (recurrent implantation failure) (blue) and male factor (grey) cervical samples in the space of the first two principal components (PC1 and PC2). The correlation between the original variables and the principal components was evaluated using the Pearson correlation coefficient. (B) Heatmap of significantly different metabolic pathways inferred by PICRUSt2 between groups in cervix. Each row represents a metabolic pathway and each column an individual sample. Z-score values, shown by the colour scale, were calculated by normalizing pathway abundances to highlight significant differences. Hierarchical clustering was performed using Euclidean distance and average linkage method. Supplementary Figure S14. Prediction of functional profiles based on KEGG database in endometrial samples. (A) Principal Coordinate Analysis (PCA) of predicted metabolic pathways using PICRUSt2. The plot shows the distribution of endometriosis (pink), RIF (recurrent implantation failure) (blue) and male factor (grey) endometrial samples in the space of the first two principal components (PC1 and PC2). The correlation between the original variables and the principal components was evaluated using the Pearson correlation coefficient. (B) Heatmap of significantly different metabolic pathways inferred by PICRUSt2 between groups in endometrium. Each row represents a metabolic pathway and each column an individual sample. Z-score values, shown by the colour scale, were calculated by normalizing pathway abundances to highlight significant differences. Hierarchical clustering was performed using Euclidean distance and average linkage method. Supplementary Figure S15. Prediction of functional profiles based on KEGG database in urine samples. (A) Principal Coordinate Analysis (PCA) of predicted metabolic pathways using PICRUSt2. The plot shows the distribution of endometriosis (pink), RIF (recurrent implantation failure) (blue) and male factor (grey) urine samples in the space of the first two principal components (PC1 and PC2). The correlation between the original variables and the principal components was evaluated using the Pearson correlation coefficient. (B) Heatmap of significantly different metabolic pathways inferred by PICRUSt2 between groups in urine. Each row represents a metabolic pathway and each column an individual sample. Z-score values, shown by the colour scale, were calculated by normalizing pathway abundances to highlight significant differences. Hierarchical clustering was performed using Euclidean distance and average linkage method. Supplementary Figure S16. Prediction of functional profiles based on KEGG database in rectum samples. (A) Principal Coordinate Analysis (PCA) of predicted metabolic pathways using PICRUSt2. The plot shows the distribution of endometriosis (pink), RIF (recurrent implantation failure) (blue) and male factor (grey) rectal samples in the space of the first two principal components (PC1 and PC2). The correlation between the original variables and the principal components was evaluated using the Pearson correlation coefficient. (B) Heatmap of significantly different metabolic pathways inferred by PICRUSt2 between groups in rectum. Each row represents a metabolic pathway and each column an individual sample. Z-score values, shown by the colour scale, were calculated by normalizing pathway abundances to highlight significant differences. Hierarchical clustering was performed using Euclidean distance and average linkage method. Supplementary Figure S17. Prediction of functional profiles based on Enzyme Commission (EC) annotations in vaginal samples. (A) Principal Coordinate Analysis (PCA) of predicted metabolic pathways using PICRUSt2. The plot shows the distribution of endometriosis (pink), RIF (recurrent implantation failure) (blue) and male factor (grey) vaginal samples in the space of the first two principal components (PC1 and PC2). The correlation between the original variables and the principal components was evaluated using the Pearson correlation coefficient. (B) Heatmap of significantly different metabolic pathways inferred by PICRUSt2 between groups in vagina. Each row represents a metabolic pathway and each column an individual sample. Z-score values, shown by the colour scale, were calculated by normalizing pathway abundances to highlight significant differences. Hierarchical clustering was performed using Euclidean distance and average linkage method. Supplementary Figure S18. Prediction of functional profiles based on Enzyme Commission (EC) annotations in cervical samples. (A) Principal Coordinate Analysis (PCA) of predicted metabolic pathways using PICRUSt2. The plot shows the distribution of endometriosis (pink), RIF (recurrent implantation failure) (blue) and male factor (grey) cervical samples in the space of the first two principal components (PC1 and PC2). The correlation between the original variables and the principal components was evaluated using the Pearson correlation coefficient. (B) Heatmap of significantly different metabolic pathways inferred by PICRUSt2 between groups in cervix. Each row represents a metabolic pathway and each column an individual sample. Z-score values, shown by the colour scale, were calculated by normalizing pathway abundances to highlight significant differences. Hierarchical clustering was performed using Euclidean distance and average linkage method. Supplementary Figure S19. Prediction of functional profiles based on Enzyme Commission (EC) annotations in endometrial samples. (A) Principal Coordinate Analysis (PCA) of predicted metabolic pathways using PICRUSt2. The plot shows the distribution of endometriosis (pink), RIF (recurrent implantation failure) (blue) and male factor (grey) endometrial samples in the space of the first two principal components (PC1 and PC2). The correlation between the original variables and the principal components was evaluated using the Pearson correlation coefficient. (B) Heatmap of significantly different metabolic pathways inferred by PICRUSt2 between groups in endometrium. Each row represents a metabolic pathway and each column an individual sample. Z-score values, shown by the colour scale, were calculated by normalizing pathway abundances to highlight significant differences. Hierarchical clustering was performed using Euclidean distance and average linkage method. Supplementary Figure S20. Prediction of functional profiles based on Enzyme Commission (EC) annotations in urine samples. (A) Principal Coordinate Analysis (PCA) of predicted metabolic pathways using PICRUSt2. The plot shows the distribution of endometriosis (pink), RIF (recurrent implantation failure) (blue) and male factor (grey) urine samples in the space of the first two principal components (PC1 and PC2). The correlation between the original variables and the principal components was evaluated using the Pearson correlation coefficient. (B) Heatmap of significantly different metabolic pathways inferred by PICRUSt2 between groups in urine. Each row represents a metabolic pathway and each column an individual sample. Z-score values, shown by the colour scale, were calculated by normalizing pathway abundances to highlight significant differences. Hierarchical clustering was performed using Euclidean distance and average linkage method. Supplementary Figure S21. Prediction of functional profiles based on Enzyme Commission (EC) annotations in rectal samples. (A) Principal Coordinate Analysis (PCA) of predicted metabolic pathways using PICRUSt2. The plot shows the distribution of endometriosis (pink), RIF (recurrent implantation failure) (blue) and male factor (grey) rectal samples in the space of the first two principal components (PC1 and PC2). The correlation between the original variables and the principal components was evaluated using the Pearson correlation coefficient. (B) Heatmap of significantly different metabolic pathways inferred by PICRUSt2 between groups in rectum. Each row represents a metabolic pathway and each column an individual sample. Z-score values, shown by the colour scale, were calculated by normalizing pathway abundances to highlight significant differences. Hierarchical clustering was performed using Euclidean distance and average linkage method.

## Data Availability

The datasets generated and analysed during the current study are available in the Sequence Read Archive (SRA) under BioProject ID: PRJNA1338101 (https:/www.ncbi.nlm.nih.gov/sra).

## Abbreviations

ANCOVA: Analysis of Covariance test
ARTs: Assisted Reproduction Techniques
ASVs: Amplicon sequence variants
BMI: Body mass index
EC: Enzyme Commission
FDR: False discovery rate
KEGG: Kyoto Encyclopedia of Genes and Genomes
LH: Luteinising hormone
LogFC: Logarithm fold change
PCA: Principal Component Analysis
PCoA: Principal Coordinates Analysis
PCOS: Polycystic ovary syndrome
PERMANOVA: Permutational Multivariate Analysis of Variance
PICRUSt2: Phylogenetic Investigation of Communities by Reconstruction of Unobserved States
QIIME2: Quantitative Insights Into Microbial Ecology version 2
RIF: Recurrent implantation failure
RPL: Recurrent pregnancy loss
SD: Standard deviation
WHO: World Health Organization
