# Supplementary figures and images for: Assessing the female urogenital-rectal axis microbiome: focus on endometriosis and recurrent implantation failure
Source: Reprod Biol Endocrinol. 2026 Jul 21;24:75. doi: 10.1186/s12958-026-01595-0 (PMC13390397; doi:10.1186/s12958-026-01595-0)

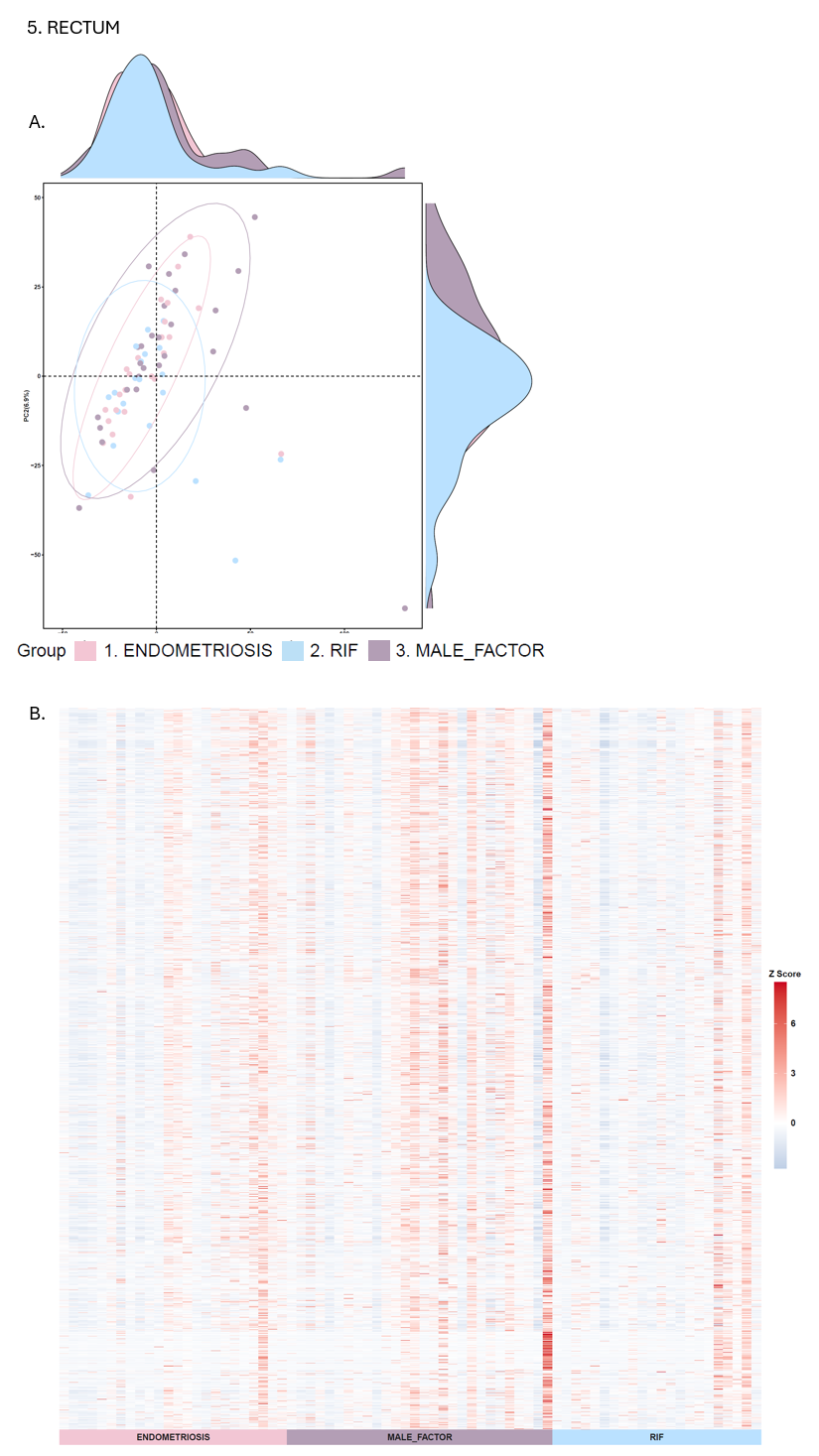

Supplement: Supplementary file 2 — Supplementary Material 2: Supplementary Figure S1. (A) Percentages of true (purple) and contaminant (grey) reads in uterus samples by participant. (B) Percentages of true (yellow) and contaminant (grey) reads in urine samples by participant. Supplementary Figure S2. Venn Diagram showing total bacterial genera identified in sensitivity analysis population. The size of the circle is proportional to the number of bacteria. (A) Bacteria shared within the female reproductive tract (vagina, cervix and uterus). (B) Bacteria shared between the female reproductive tract (vagina, cervix and uterus) and adjacent sites (urine and rectum). Supplementary Figure S3. Dot-boxplot of the relative abundance of Lactobacillus in each body site. Each dot represents the relative abundance of Lactobacillus of each participant in sensitivity population. The bold line within the box shows the median for each group. Supplementary Figure S4. Microbial composition across the body sites in sensitivity population. Iris plots represent those bacterial genera with a relative abundance > 1%. The bacterial genera whose relative abundance were <1% were grouped together and labelled as “Other”. Supplementary Figure S5. Diversity analysis in sensitivity population (A, B) Alpha-diversity evaluated by Shannon diversity index and Richness, respectively. (C) Beta-diversity represented using a principal coordinate analysis (PCoA) based on the Bray-Curtis distance. Supplementary Figure S6. Correlation analysis of microbial abundance between vagina, cervix and uterus in sensitivity population. Heatmaps represent those common bacterial genera between sites with a relative abundance > 1%. Associations were performed based on Spearman’s correlation. Positive correlations are displayed in red and negative correlations in blue. Colour intensity and the size of the circles are proportional to the correlation coefficients. Correlation results were considered statistically significant according to the following p-valu [file 12958_2026_1595_MOESM2_ESM.zip › Supplementary_figure_S21.png]

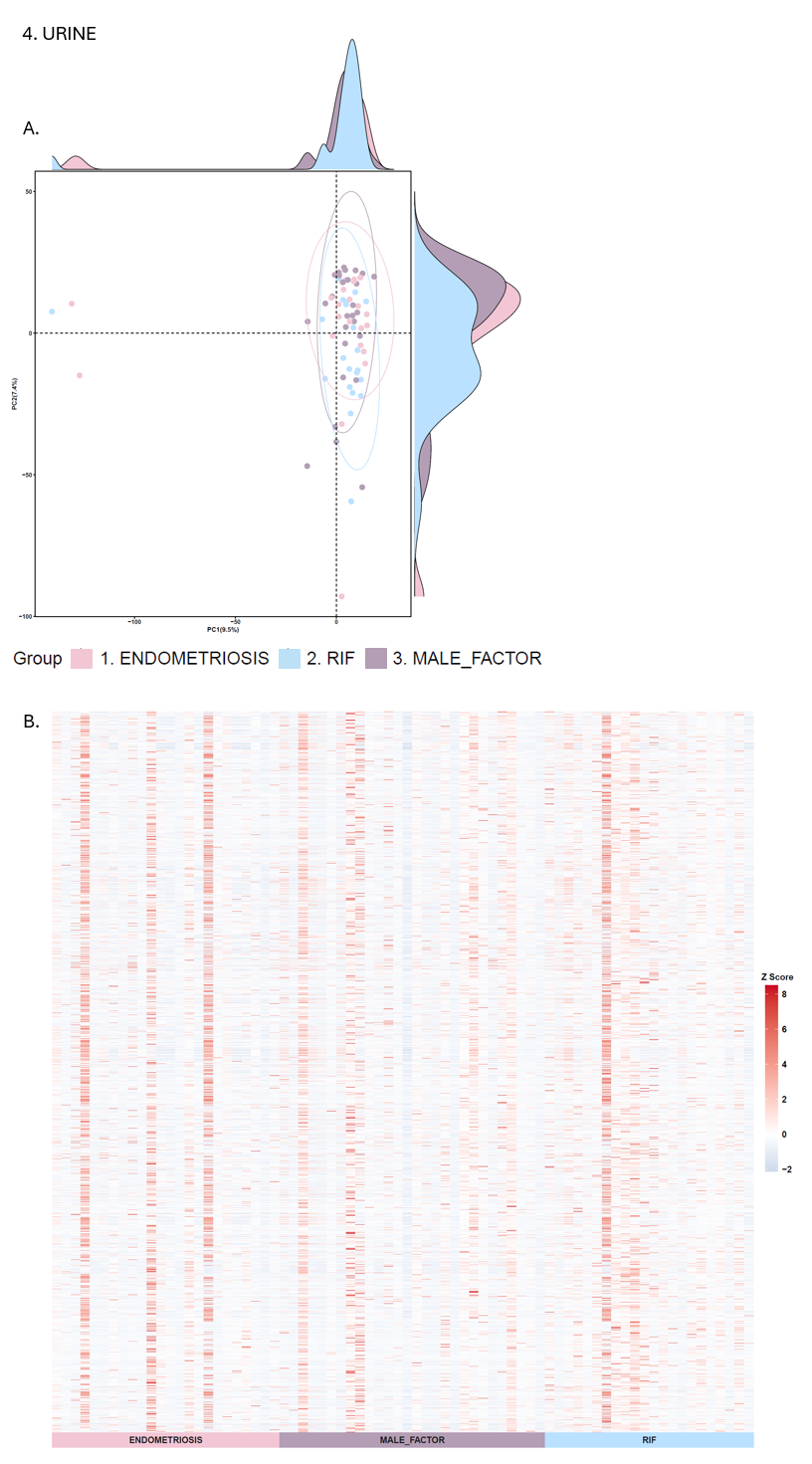

Supplement: Supplementary file 2 — Supplementary Material 2: Supplementary Figure S1. (A) Percentages of true (purple) and contaminant (grey) reads in uterus samples by participant. (B) Percentages of true (yellow) and contaminant (grey) reads in urine samples by participant. Supplementary Figure S2. Venn Diagram showing total bacterial genera identified in sensitivity analysis population. The size of the circle is proportional to the number of bacteria. (A) Bacteria shared within the female reproductive tract (vagina, cervix and uterus). (B) Bacteria shared between the female reproductive tract (vagina, cervix and uterus) and adjacent sites (urine and rectum). Supplementary Figure S3. Dot-boxplot of the relative abundance of Lactobacillus in each body site. Each dot represents the relative abundance of Lactobacillus of each participant in sensitivity population. The bold line within the box shows the median for each group. Supplementary Figure S4. Microbial composition across the body sites in sensitivity population. Iris plots represent those bacterial genera with a relative abundance > 1%. The bacterial genera whose relative abundance were <1% were grouped together and labelled as “Other”. Supplementary Figure S5. Diversity analysis in sensitivity population (A, B) Alpha-diversity evaluated by Shannon diversity index and Richness, respectively. (C) Beta-diversity represented using a principal coordinate analysis (PCoA) based on the Bray-Curtis distance. Supplementary Figure S6. Correlation analysis of microbial abundance between vagina, cervix and uterus in sensitivity population. Heatmaps represent those common bacterial genera between sites with a relative abundance > 1%. Associations were performed based on Spearman’s correlation. Positive correlations are displayed in red and negative correlations in blue. Colour intensity and the size of the circles are proportional to the correlation coefficients. Correlation results were considered statistically significant according to the following p-valu [file 12958_2026_1595_MOESM2_ESM.zip › Supplementary_figure_S20.png]

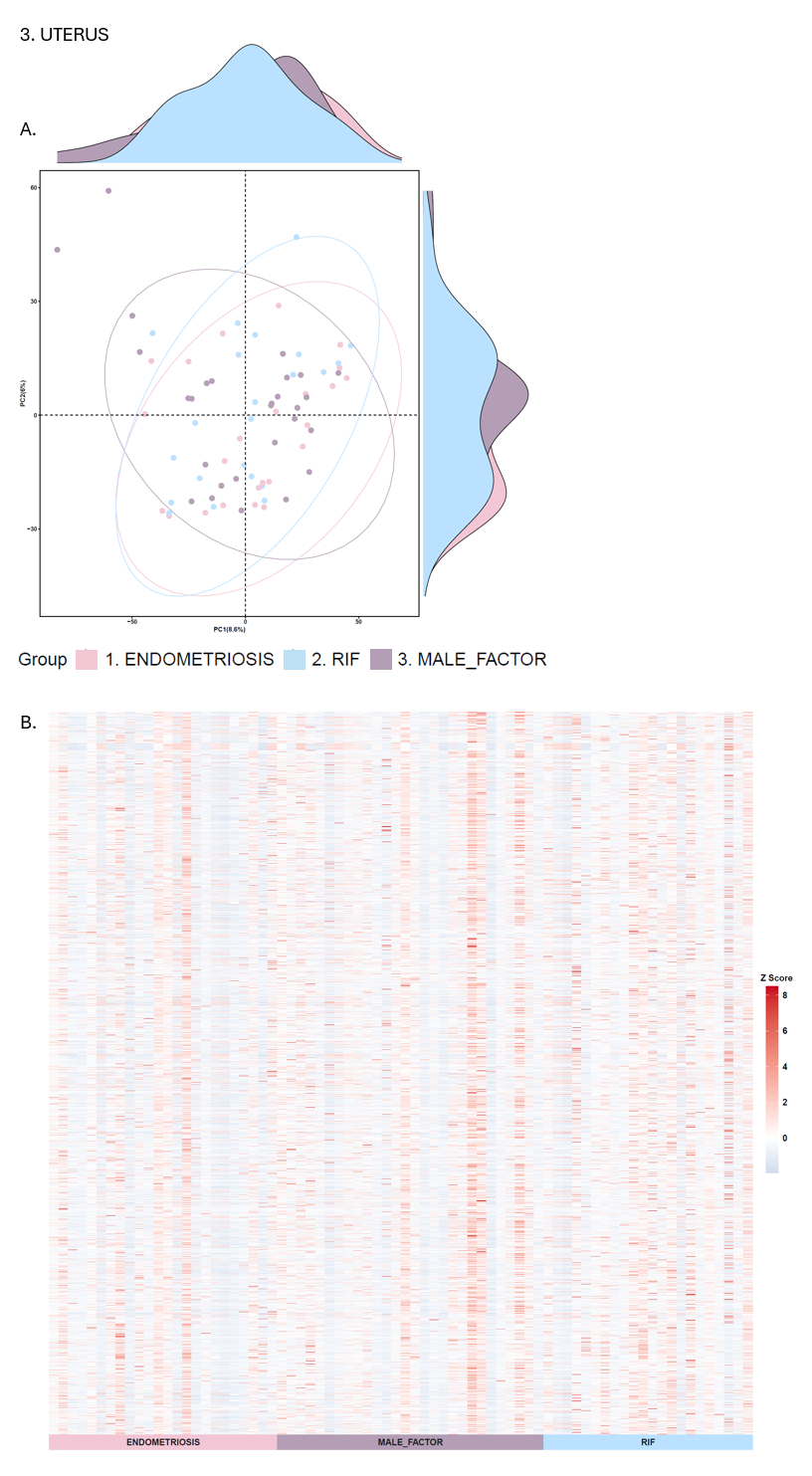

Supplement: Supplementary file 2 — Supplementary Material 2: Supplementary Figure S1. (A) Percentages of true (purple) and contaminant (grey) reads in uterus samples by participant. (B) Percentages of true (yellow) and contaminant (grey) reads in urine samples by participant. Supplementary Figure S2. Venn Diagram showing total bacterial genera identified in sensitivity analysis population. The size of the circle is proportional to the number of bacteria. (A) Bacteria shared within the female reproductive tract (vagina, cervix and uterus). (B) Bacteria shared between the female reproductive tract (vagina, cervix and uterus) and adjacent sites (urine and rectum). Supplementary Figure S3. Dot-boxplot of the relative abundance of Lactobacillus in each body site. Each dot represents the relative abundance of Lactobacillus of each participant in sensitivity population. The bold line within the box shows the median for each group. Supplementary Figure S4. Microbial composition across the body sites in sensitivity population. Iris plots represent those bacterial genera with a relative abundance > 1%. The bacterial genera whose relative abundance were <1% were grouped together and labelled as “Other”. Supplementary Figure S5. Diversity analysis in sensitivity population (A, B) Alpha-diversity evaluated by Shannon diversity index and Richness, respectively. (C) Beta-diversity represented using a principal coordinate analysis (PCoA) based on the Bray-Curtis distance. Supplementary Figure S6. Correlation analysis of microbial abundance between vagina, cervix and uterus in sensitivity population. Heatmaps represent those common bacterial genera between sites with a relative abundance > 1%. Associations were performed based on Spearman’s correlation. Positive correlations are displayed in red and negative correlations in blue. Colour intensity and the size of the circles are proportional to the correlation coefficients. Correlation results were considered statistically significant according to the following p-valu [file 12958_2026_1595_MOESM2_ESM.zip › Supplementary_figure_S19.png]

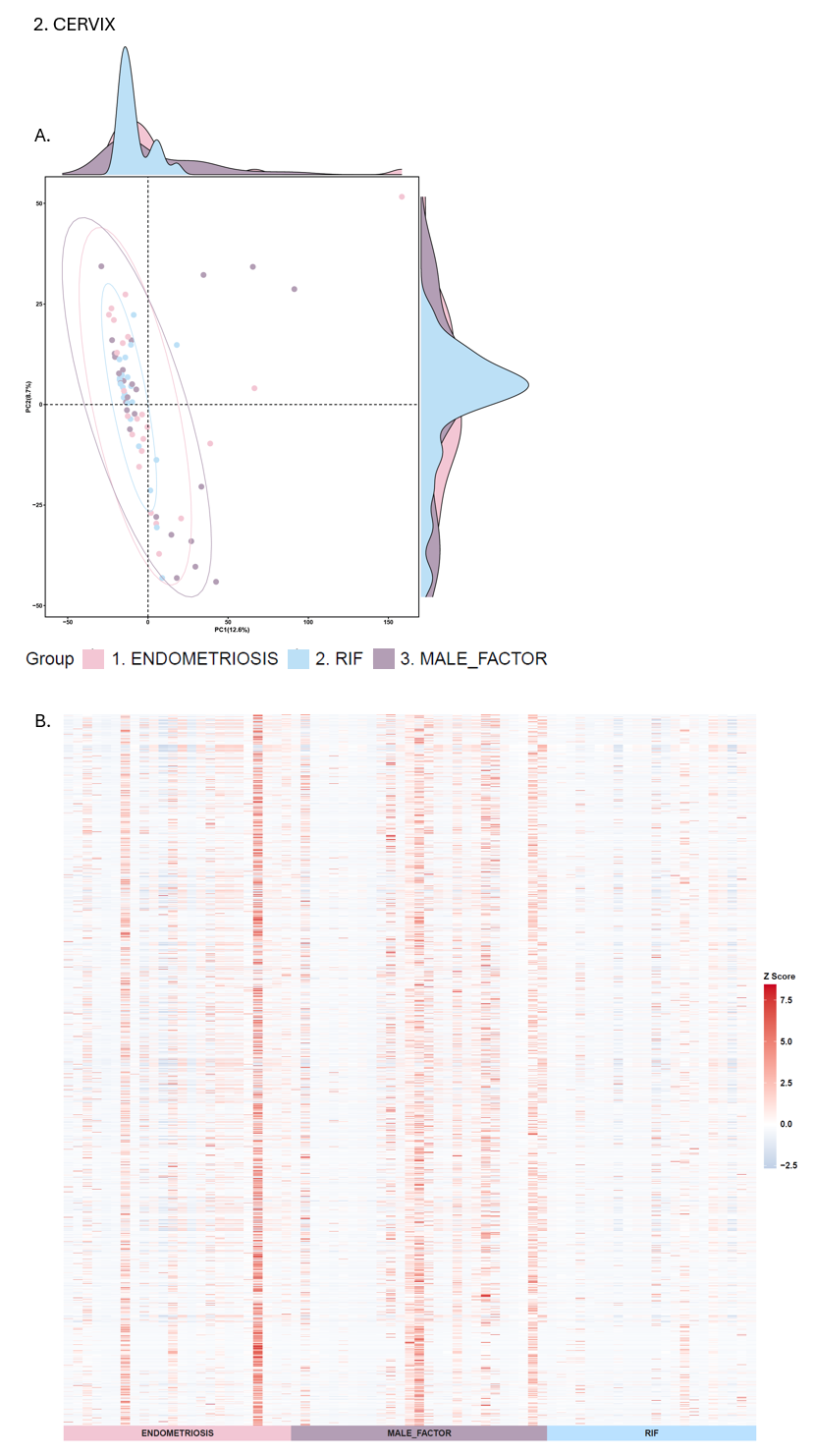

Supplement: Supplementary file 2 — Supplementary Material 2: Supplementary Figure S1. (A) Percentages of true (purple) and contaminant (grey) reads in uterus samples by participant. (B) Percentages of true (yellow) and contaminant (grey) reads in urine samples by participant. Supplementary Figure S2. Venn Diagram showing total bacterial genera identified in sensitivity analysis population. The size of the circle is proportional to the number of bacteria. (A) Bacteria shared within the female reproductive tract (vagina, cervix and uterus). (B) Bacteria shared between the female reproductive tract (vagina, cervix and uterus) and adjacent sites (urine and rectum). Supplementary Figure S3. Dot-boxplot of the relative abundance of Lactobacillus in each body site. Each dot represents the relative abundance of Lactobacillus of each participant in sensitivity population. The bold line within the box shows the median for each group. Supplementary Figure S4. Microbial composition across the body sites in sensitivity population. Iris plots represent those bacterial genera with a relative abundance > 1%. The bacterial genera whose relative abundance were <1% were grouped together and labelled as “Other”. Supplementary Figure S5. Diversity analysis in sensitivity population (A, B) Alpha-diversity evaluated by Shannon diversity index and Richness, respectively. (C) Beta-diversity represented using a principal coordinate analysis (PCoA) based on the Bray-Curtis distance. Supplementary Figure S6. Correlation analysis of microbial abundance between vagina, cervix and uterus in sensitivity population. Heatmaps represent those common bacterial genera between sites with a relative abundance > 1%. Associations were performed based on Spearman’s correlation. Positive correlations are displayed in red and negative correlations in blue. Colour intensity and the size of the circles are proportional to the correlation coefficients. Correlation results were considered statistically significant according to the following p-valu [file 12958_2026_1595_MOESM2_ESM.zip › Supplementary_figure_S18.png]

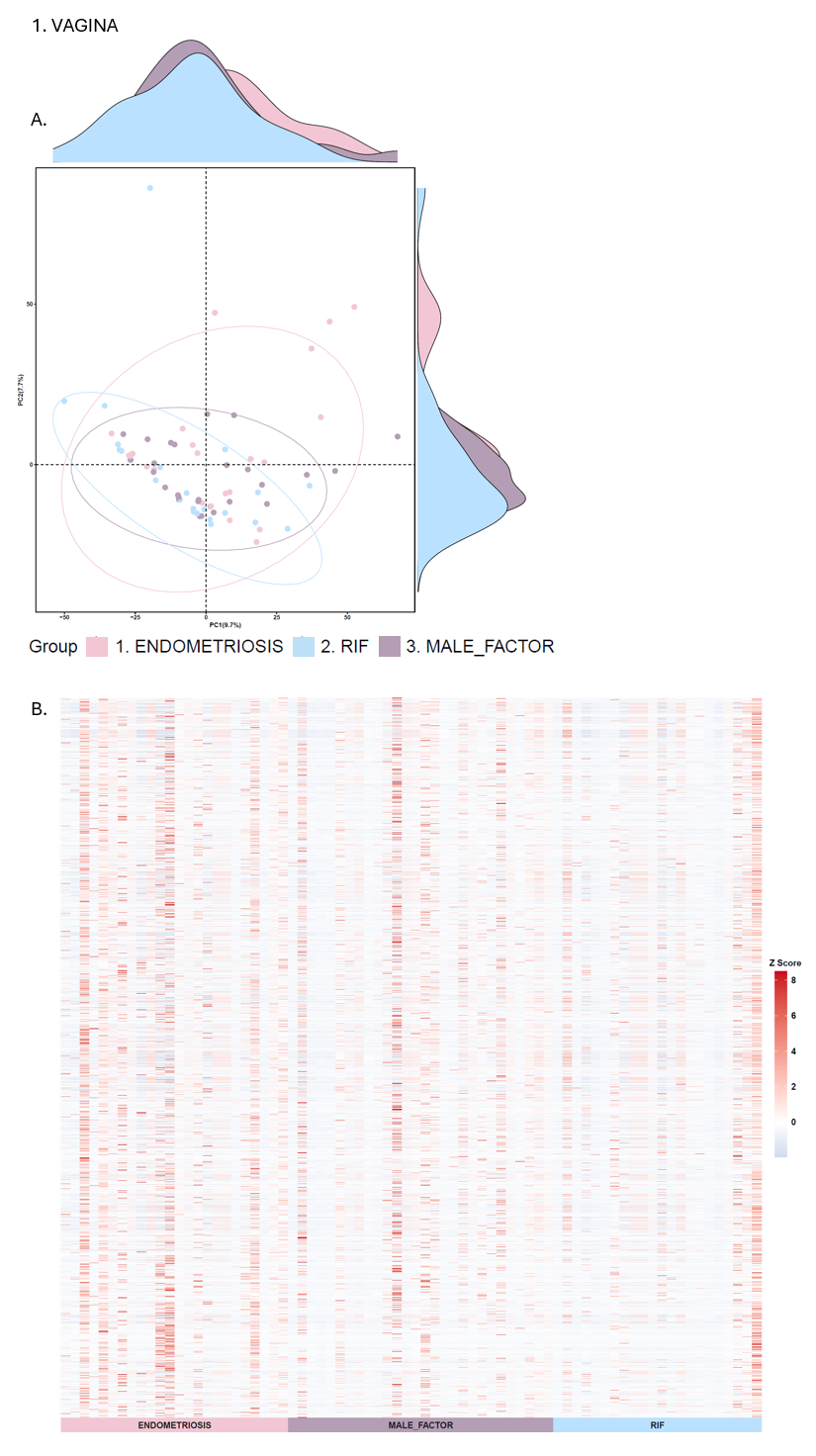

Supplement: Supplementary file 2 — Supplementary Material 2: Supplementary Figure S1. (A) Percentages of true (purple) and contaminant (grey) reads in uterus samples by participant. (B) Percentages of true (yellow) and contaminant (grey) reads in urine samples by participant. Supplementary Figure S2. Venn Diagram showing total bacterial genera identified in sensitivity analysis population. The size of the circle is proportional to the number of bacteria. (A) Bacteria shared within the female reproductive tract (vagina, cervix and uterus). (B) Bacteria shared between the female reproductive tract (vagina, cervix and uterus) and adjacent sites (urine and rectum). Supplementary Figure S3. Dot-boxplot of the relative abundance of Lactobacillus in each body site. Each dot represents the relative abundance of Lactobacillus of each participant in sensitivity population. The bold line within the box shows the median for each group. Supplementary Figure S4. Microbial composition across the body sites in sensitivity population. Iris plots represent those bacterial genera with a relative abundance > 1%. The bacterial genera whose relative abundance were <1% were grouped together and labelled as “Other”. Supplementary Figure S5. Diversity analysis in sensitivity population (A, B) Alpha-diversity evaluated by Shannon diversity index and Richness, respectively. (C) Beta-diversity represented using a principal coordinate analysis (PCoA) based on the Bray-Curtis distance. Supplementary Figure S6. Correlation analysis of microbial abundance between vagina, cervix and uterus in sensitivity population. Heatmaps represent those common bacterial genera between sites with a relative abundance > 1%. Associations were performed based on Spearman’s correlation. Positive correlations are displayed in red and negative correlations in blue. Colour intensity and the size of the circles are proportional to the correlation coefficients. Correlation results were considered statistically significant according to the following p-valu [file 12958_2026_1595_MOESM2_ESM.zip › Supplementary_figure_S17.png]

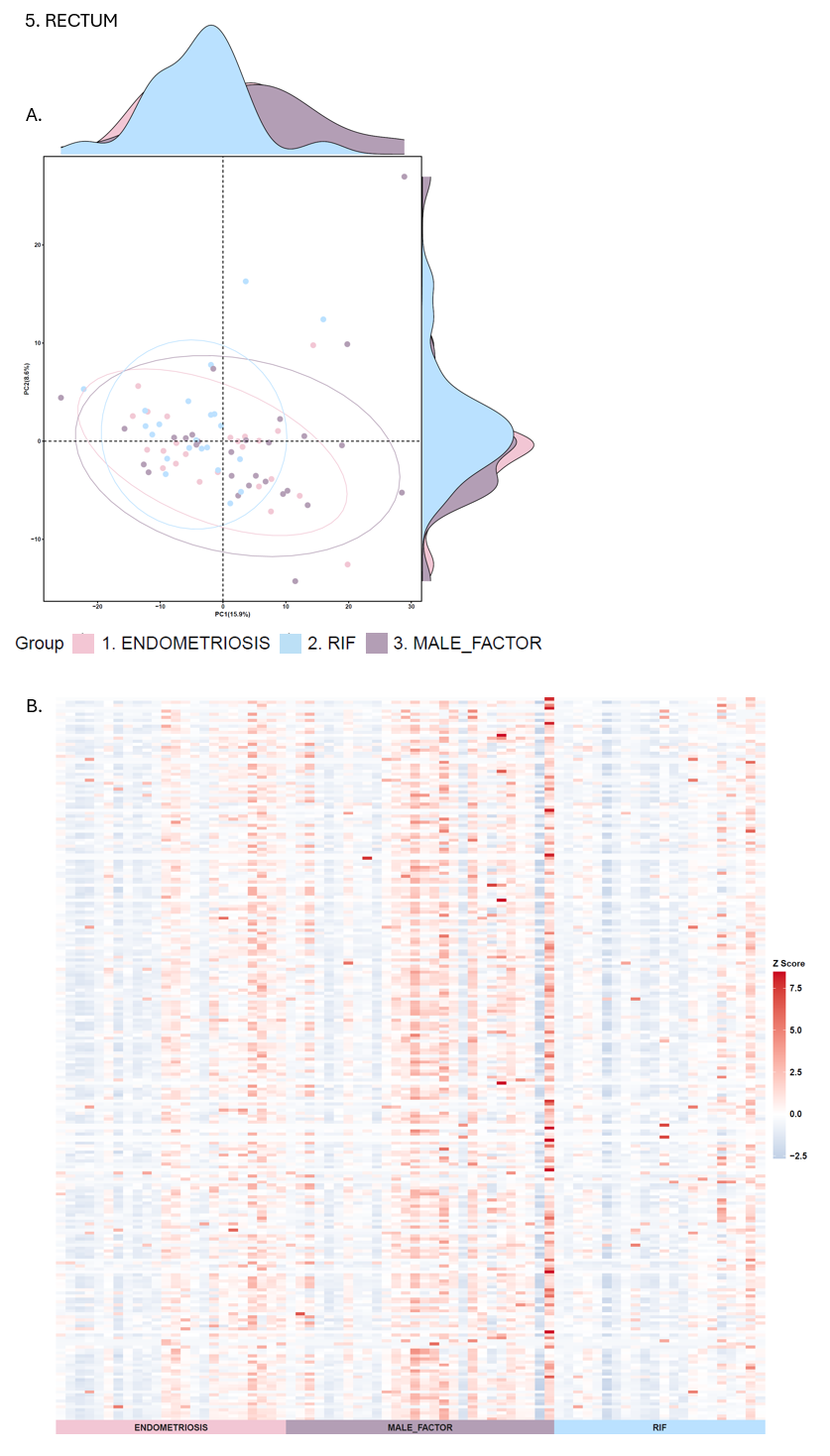

Supplement: Supplementary file 2 — Supplementary Material 2: Supplementary Figure S1. (A) Percentages of true (purple) and contaminant (grey) reads in uterus samples by participant. (B) Percentages of true (yellow) and contaminant (grey) reads in urine samples by participant. Supplementary Figure S2. Venn Diagram showing total bacterial genera identified in sensitivity analysis population. The size of the circle is proportional to the number of bacteria. (A) Bacteria shared within the female reproductive tract (vagina, cervix and uterus). (B) Bacteria shared between the female reproductive tract (vagina, cervix and uterus) and adjacent sites (urine and rectum). Supplementary Figure S3. Dot-boxplot of the relative abundance of Lactobacillus in each body site. Each dot represents the relative abundance of Lactobacillus of each participant in sensitivity population. The bold line within the box shows the median for each group. Supplementary Figure S4. Microbial composition across the body sites in sensitivity population. Iris plots represent those bacterial genera with a relative abundance > 1%. The bacterial genera whose relative abundance were <1% were grouped together and labelled as “Other”. Supplementary Figure S5. Diversity analysis in sensitivity population (A, B) Alpha-diversity evaluated by Shannon diversity index and Richness, respectively. (C) Beta-diversity represented using a principal coordinate analysis (PCoA) based on the Bray-Curtis distance. Supplementary Figure S6. Correlation analysis of microbial abundance between vagina, cervix and uterus in sensitivity population. Heatmaps represent those common bacterial genera between sites with a relative abundance > 1%. Associations were performed based on Spearman’s correlation. Positive correlations are displayed in red and negative correlations in blue. Colour intensity and the size of the circles are proportional to the correlation coefficients. Correlation results were considered statistically significant according to the following p-valu [file 12958_2026_1595_MOESM2_ESM.zip › Supplementary_figure_S16.png]

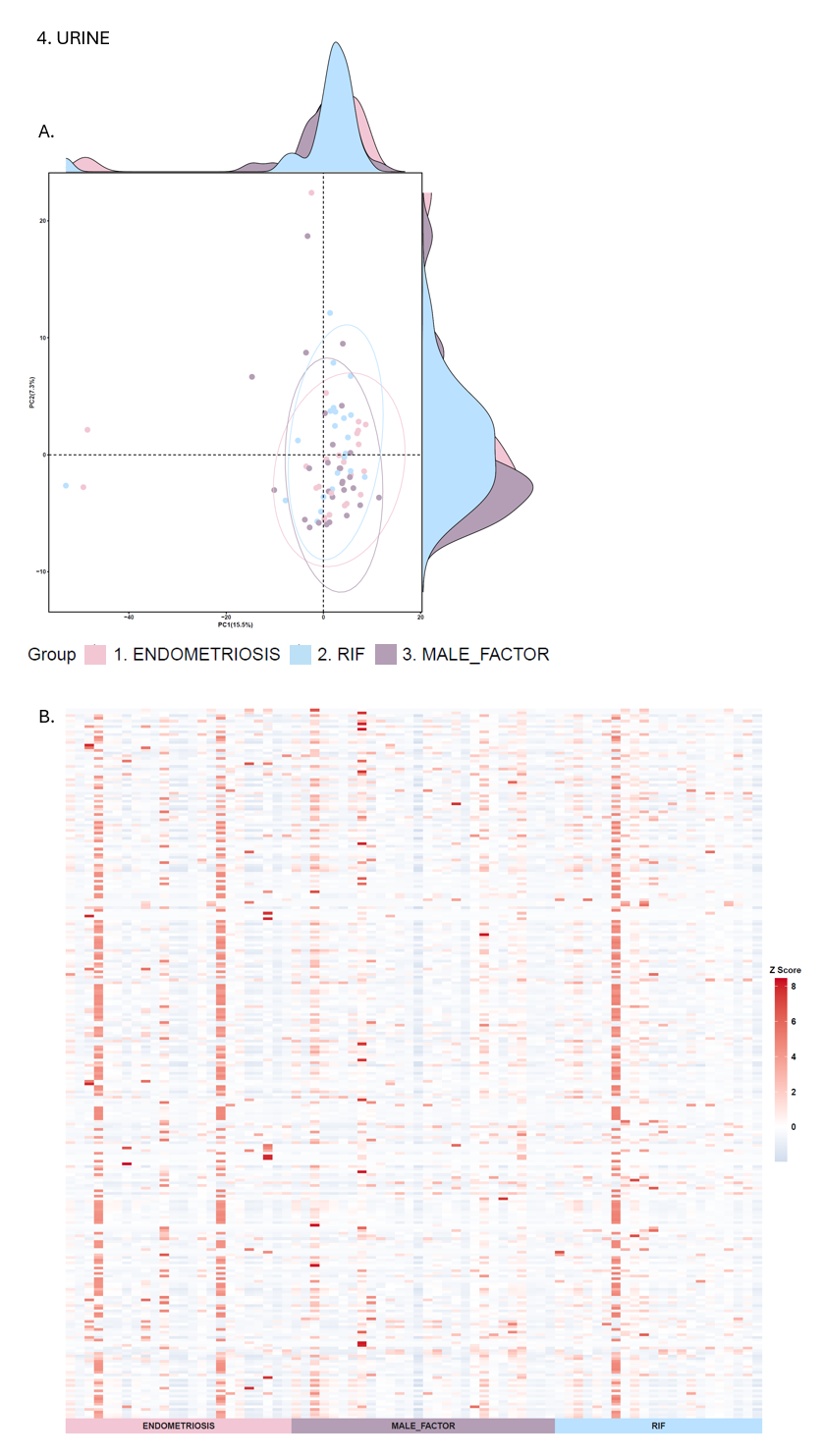

Supplement: Supplementary file 2 — Supplementary Material 2: Supplementary Figure S1. (A) Percentages of true (purple) and contaminant (grey) reads in uterus samples by participant. (B) Percentages of true (yellow) and contaminant (grey) reads in urine samples by participant. Supplementary Figure S2. Venn Diagram showing total bacterial genera identified in sensitivity analysis population. The size of the circle is proportional to the number of bacteria. (A) Bacteria shared within the female reproductive tract (vagina, cervix and uterus). (B) Bacteria shared between the female reproductive tract (vagina, cervix and uterus) and adjacent sites (urine and rectum). Supplementary Figure S3. Dot-boxplot of the relative abundance of Lactobacillus in each body site. Each dot represents the relative abundance of Lactobacillus of each participant in sensitivity population. The bold line within the box shows the median for each group. Supplementary Figure S4. Microbial composition across the body sites in sensitivity population. Iris plots represent those bacterial genera with a relative abundance > 1%. The bacterial genera whose relative abundance were <1% were grouped together and labelled as “Other”. Supplementary Figure S5. Diversity analysis in sensitivity population (A, B) Alpha-diversity evaluated by Shannon diversity index and Richness, respectively. (C) Beta-diversity represented using a principal coordinate analysis (PCoA) based on the Bray-Curtis distance. Supplementary Figure S6. Correlation analysis of microbial abundance between vagina, cervix and uterus in sensitivity population. Heatmaps represent those common bacterial genera between sites with a relative abundance > 1%. Associations were performed based on Spearman’s correlation. Positive correlations are displayed in red and negative correlations in blue. Colour intensity and the size of the circles are proportional to the correlation coefficients. Correlation results were considered statistically significant according to the following p-valu [file 12958_2026_1595_MOESM2_ESM.zip › Supplementary_figure_S15.png]

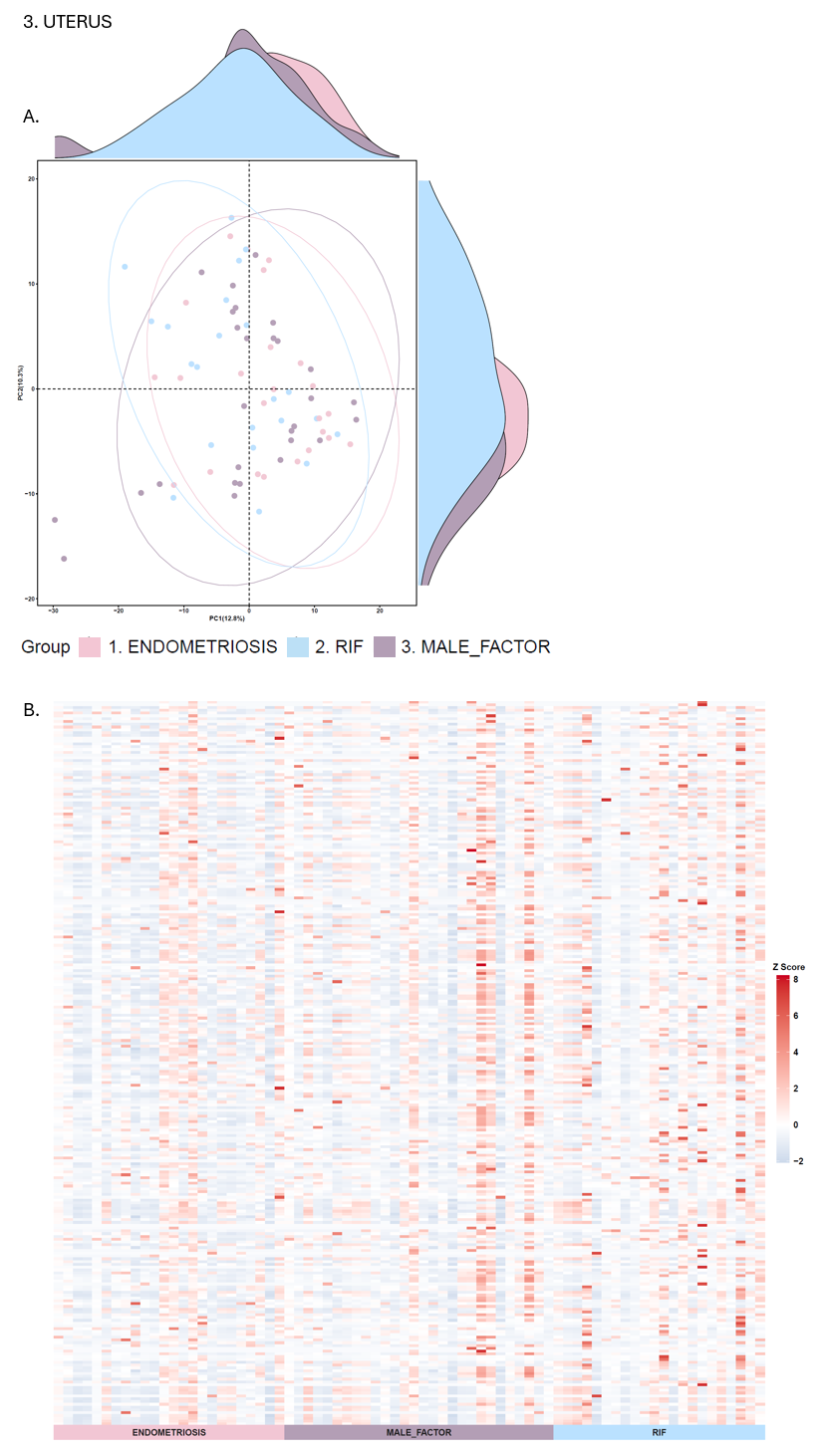

Supplement: Supplementary file 2 — Supplementary Material 2: Supplementary Figure S1. (A) Percentages of true (purple) and contaminant (grey) reads in uterus samples by participant. (B) Percentages of true (yellow) and contaminant (grey) reads in urine samples by participant. Supplementary Figure S2. Venn Diagram showing total bacterial genera identified in sensitivity analysis population. The size of the circle is proportional to the number of bacteria. (A) Bacteria shared within the female reproductive tract (vagina, cervix and uterus). (B) Bacteria shared between the female reproductive tract (vagina, cervix and uterus) and adjacent sites (urine and rectum). Supplementary Figure S3. Dot-boxplot of the relative abundance of Lactobacillus in each body site. Each dot represents the relative abundance of Lactobacillus of each participant in sensitivity population. The bold line within the box shows the median for each group. Supplementary Figure S4. Microbial composition across the body sites in sensitivity population. Iris plots represent those bacterial genera with a relative abundance > 1%. The bacterial genera whose relative abundance were <1% were grouped together and labelled as “Other”. Supplementary Figure S5. Diversity analysis in sensitivity population (A, B) Alpha-diversity evaluated by Shannon diversity index and Richness, respectively. (C) Beta-diversity represented using a principal coordinate analysis (PCoA) based on the Bray-Curtis distance. Supplementary Figure S6. Correlation analysis of microbial abundance between vagina, cervix and uterus in sensitivity population. Heatmaps represent those common bacterial genera between sites with a relative abundance > 1%. Associations were performed based on Spearman’s correlation. Positive correlations are displayed in red and negative correlations in blue. Colour intensity and the size of the circles are proportional to the correlation coefficients. Correlation results were considered statistically significant according to the following p-valu [file 12958_2026_1595_MOESM2_ESM.zip › Supplementary_figure_S14.png]

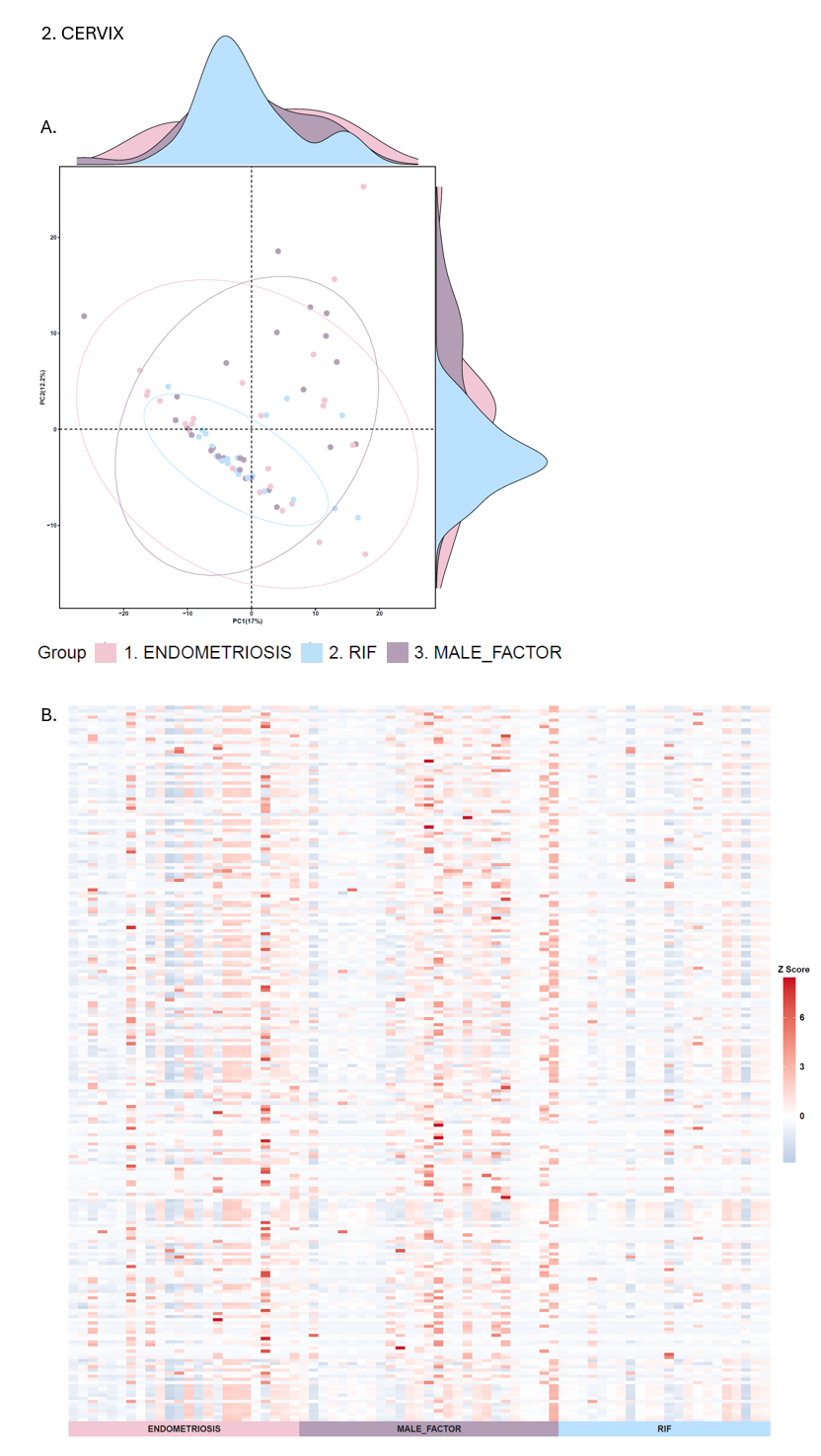

Supplement: Supplementary file 2 — Supplementary Material 2: Supplementary Figure S1. (A) Percentages of true (purple) and contaminant (grey) reads in uterus samples by participant. (B) Percentages of true (yellow) and contaminant (grey) reads in urine samples by participant. Supplementary Figure S2. Venn Diagram showing total bacterial genera identified in sensitivity analysis population. The size of the circle is proportional to the number of bacteria. (A) Bacteria shared within the female reproductive tract (vagina, cervix and uterus). (B) Bacteria shared between the female reproductive tract (vagina, cervix and uterus) and adjacent sites (urine and rectum). Supplementary Figure S3. Dot-boxplot of the relative abundance of Lactobacillus in each body site. Each dot represents the relative abundance of Lactobacillus of each participant in sensitivity population. The bold line within the box shows the median for each group. Supplementary Figure S4. Microbial composition across the body sites in sensitivity population. Iris plots represent those bacterial genera with a relative abundance > 1%. The bacterial genera whose relative abundance were <1% were grouped together and labelled as “Other”. Supplementary Figure S5. Diversity analysis in sensitivity population (A, B) Alpha-diversity evaluated by Shannon diversity index and Richness, respectively. (C) Beta-diversity represented using a principal coordinate analysis (PCoA) based on the Bray-Curtis distance. Supplementary Figure S6. Correlation analysis of microbial abundance between vagina, cervix and uterus in sensitivity population. Heatmaps represent those common bacterial genera between sites with a relative abundance > 1%. Associations were performed based on Spearman’s correlation. Positive correlations are displayed in red and negative correlations in blue. Colour intensity and the size of the circles are proportional to the correlation coefficients. Correlation results were considered statistically significant according to the following p-valu [file 12958_2026_1595_MOESM2_ESM.zip › Supplementary_figure_S13.png]

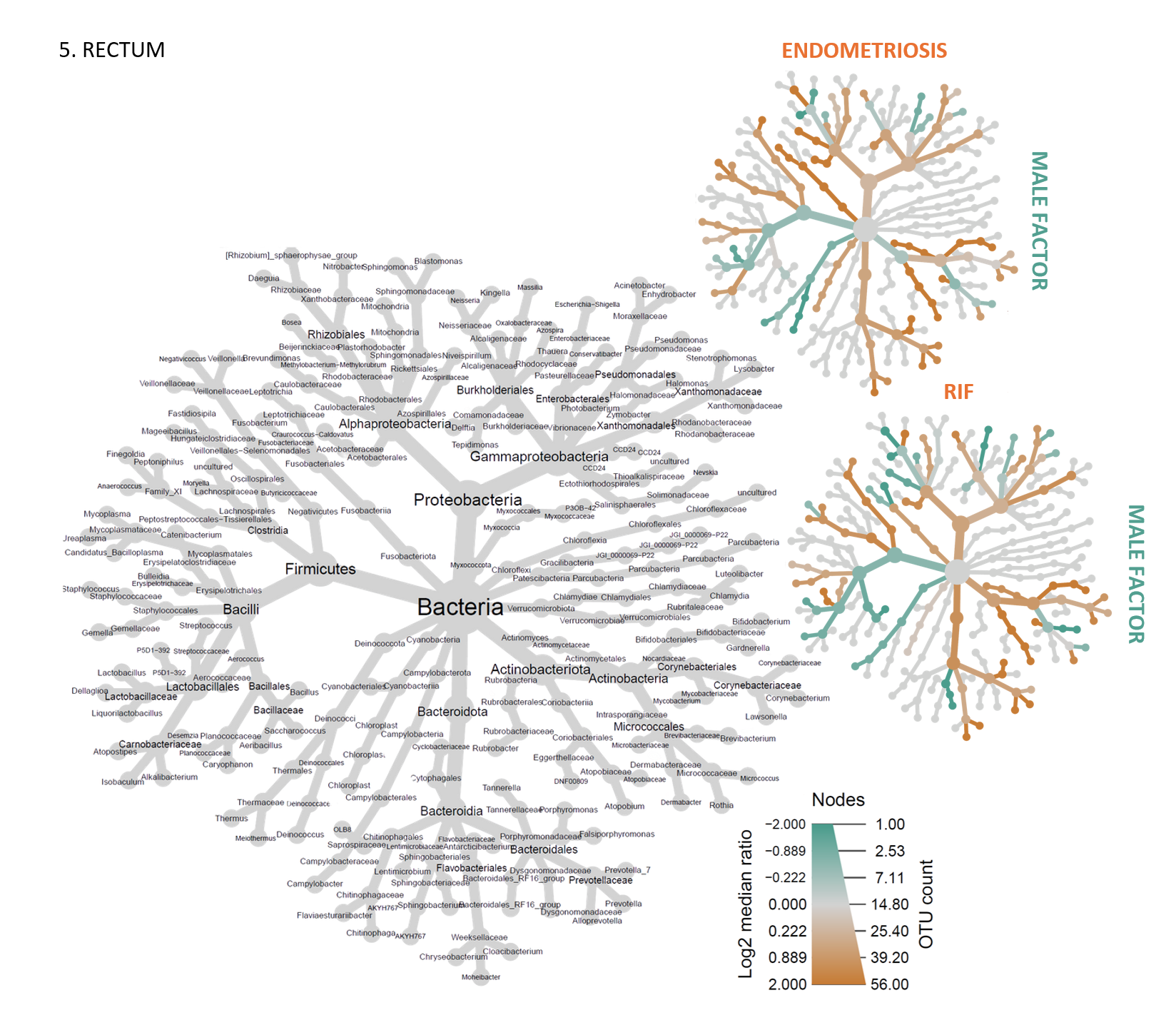

Supplement: Supplementary file 2 — Supplementary Material 2: Supplementary Figure S1. (A) Percentages of true (purple) and contaminant (grey) reads in uterus samples by participant. (B) Percentages of true (yellow) and contaminant (grey) reads in urine samples by participant. Supplementary Figure S2. Venn Diagram showing total bacterial genera identified in sensitivity analysis population. The size of the circle is proportional to the number of bacteria. (A) Bacteria shared within the female reproductive tract (vagina, cervix and uterus). (B) Bacteria shared between the female reproductive tract (vagina, cervix and uterus) and adjacent sites (urine and rectum). Supplementary Figure S3. Dot-boxplot of the relative abundance of Lactobacillus in each body site. Each dot represents the relative abundance of Lactobacillus of each participant in sensitivity population. The bold line within the box shows the median for each group. Supplementary Figure S4. Microbial composition across the body sites in sensitivity population. Iris plots represent those bacterial genera with a relative abundance > 1%. The bacterial genera whose relative abundance were <1% were grouped together and labelled as “Other”. Supplementary Figure S5. Diversity analysis in sensitivity population (A, B) Alpha-diversity evaluated by Shannon diversity index and Richness, respectively. (C) Beta-diversity represented using a principal coordinate analysis (PCoA) based on the Bray-Curtis distance. Supplementary Figure S6. Correlation analysis of microbial abundance between vagina, cervix and uterus in sensitivity population. Heatmaps represent those common bacterial genera between sites with a relative abundance > 1%. Associations were performed based on Spearman’s correlation. Positive correlations are displayed in red and negative correlations in blue. Colour intensity and the size of the circles are proportional to the correlation coefficients. Correlation results were considered statistically significant according to the following p-valu [file 12958_2026_1595_MOESM2_ESM.zip › Supplementary_figure_S12.png]

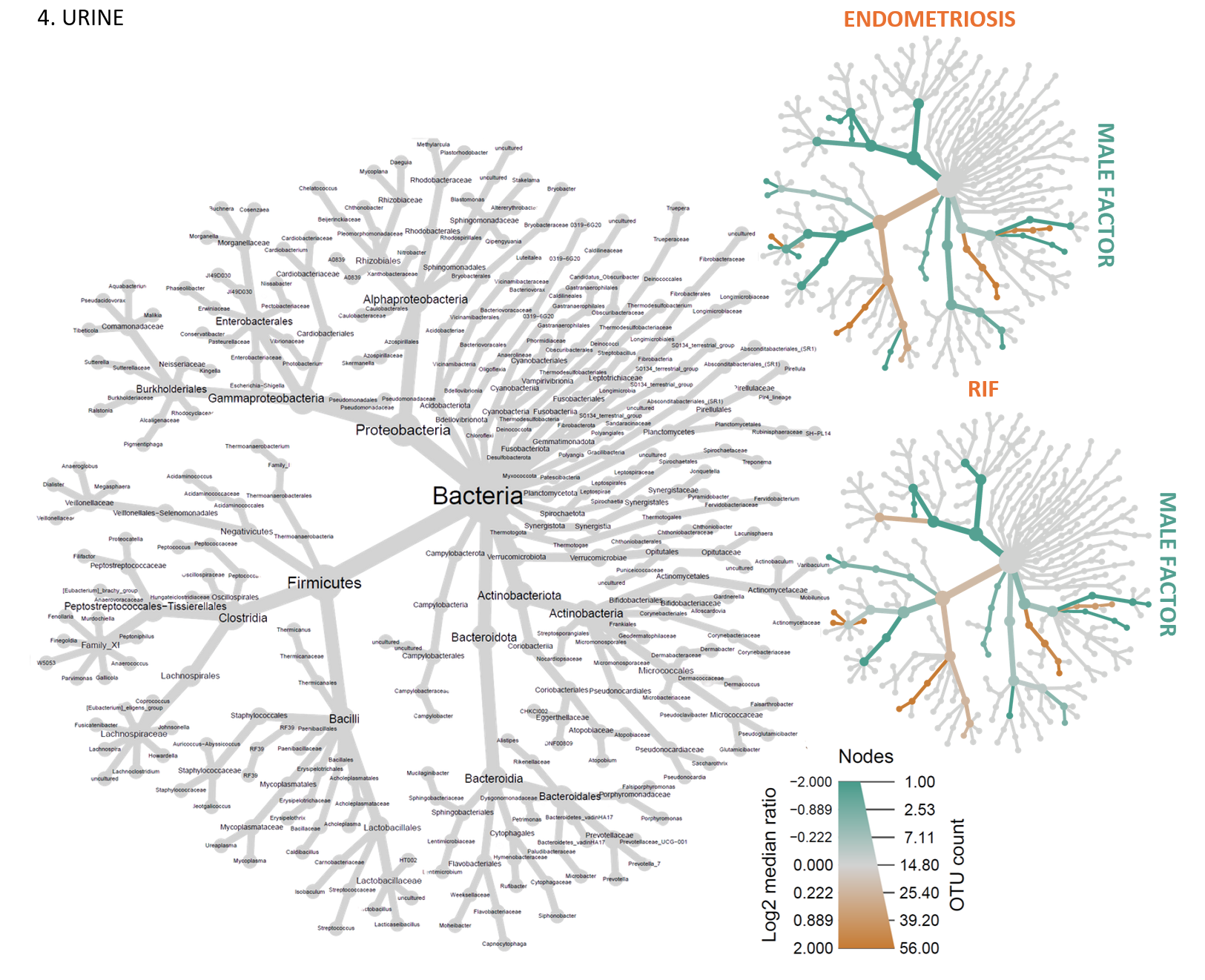

Supplement: Supplementary file 2 — Supplementary Material 2: Supplementary Figure S1. (A) Percentages of true (purple) and contaminant (grey) reads in uterus samples by participant. (B) Percentages of true (yellow) and contaminant (grey) reads in urine samples by participant. Supplementary Figure S2. Venn Diagram showing total bacterial genera identified in sensitivity analysis population. The size of the circle is proportional to the number of bacteria. (A) Bacteria shared within the female reproductive tract (vagina, cervix and uterus). (B) Bacteria shared between the female reproductive tract (vagina, cervix and uterus) and adjacent sites (urine and rectum). Supplementary Figure S3. Dot-boxplot of the relative abundance of Lactobacillus in each body site. Each dot represents the relative abundance of Lactobacillus of each participant in sensitivity population. The bold line within the box shows the median for each group. Supplementary Figure S4. Microbial composition across the body sites in sensitivity population. Iris plots represent those bacterial genera with a relative abundance > 1%. The bacterial genera whose relative abundance were <1% were grouped together and labelled as “Other”. Supplementary Figure S5. Diversity analysis in sensitivity population (A, B) Alpha-diversity evaluated by Shannon diversity index and Richness, respectively. (C) Beta-diversity represented using a principal coordinate analysis (PCoA) based on the Bray-Curtis distance. Supplementary Figure S6. Correlation analysis of microbial abundance between vagina, cervix and uterus in sensitivity population. Heatmaps represent those common bacterial genera between sites with a relative abundance > 1%. Associations were performed based on Spearman’s correlation. Positive correlations are displayed in red and negative correlations in blue. Colour intensity and the size of the circles are proportional to the correlation coefficients. Correlation results were considered statistically significant according to the following p-valu [file 12958_2026_1595_MOESM2_ESM.zip › Supplementary_figure_S11.png]

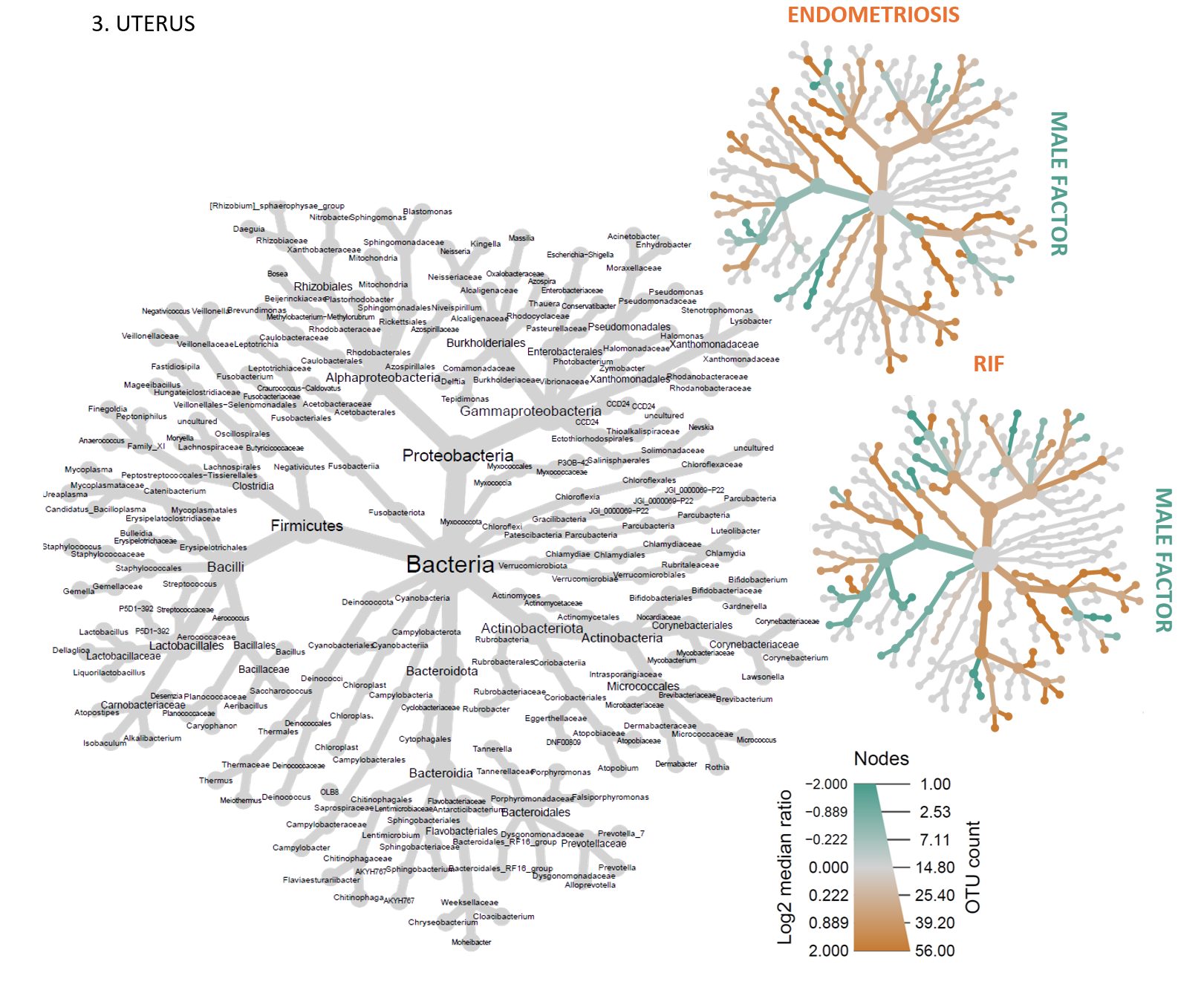

Supplement: Supplementary file 2 — Supplementary Material 2: Supplementary Figure S1. (A) Percentages of true (purple) and contaminant (grey) reads in uterus samples by participant. (B) Percentages of true (yellow) and contaminant (grey) reads in urine samples by participant. Supplementary Figure S2. Venn Diagram showing total bacterial genera identified in sensitivity analysis population. The size of the circle is proportional to the number of bacteria. (A) Bacteria shared within the female reproductive tract (vagina, cervix and uterus). (B) Bacteria shared between the female reproductive tract (vagina, cervix and uterus) and adjacent sites (urine and rectum). Supplementary Figure S3. Dot-boxplot of the relative abundance of Lactobacillus in each body site. Each dot represents the relative abundance of Lactobacillus of each participant in sensitivity population. The bold line within the box shows the median for each group. Supplementary Figure S4. Microbial composition across the body sites in sensitivity population. Iris plots represent those bacterial genera with a relative abundance > 1%. The bacterial genera whose relative abundance were <1% were grouped together and labelled as “Other”. Supplementary Figure S5. Diversity analysis in sensitivity population (A, B) Alpha-diversity evaluated by Shannon diversity index and Richness, respectively. (C) Beta-diversity represented using a principal coordinate analysis (PCoA) based on the Bray-Curtis distance. Supplementary Figure S6. Correlation analysis of microbial abundance between vagina, cervix and uterus in sensitivity population. Heatmaps represent those common bacterial genera between sites with a relative abundance > 1%. Associations were performed based on Spearman’s correlation. Positive correlations are displayed in red and negative correlations in blue. Colour intensity and the size of the circles are proportional to the correlation coefficients. Correlation results were considered statistically significant according to the following p-valu [file 12958_2026_1595_MOESM2_ESM.zip › Supplementary_figure_S10.png]

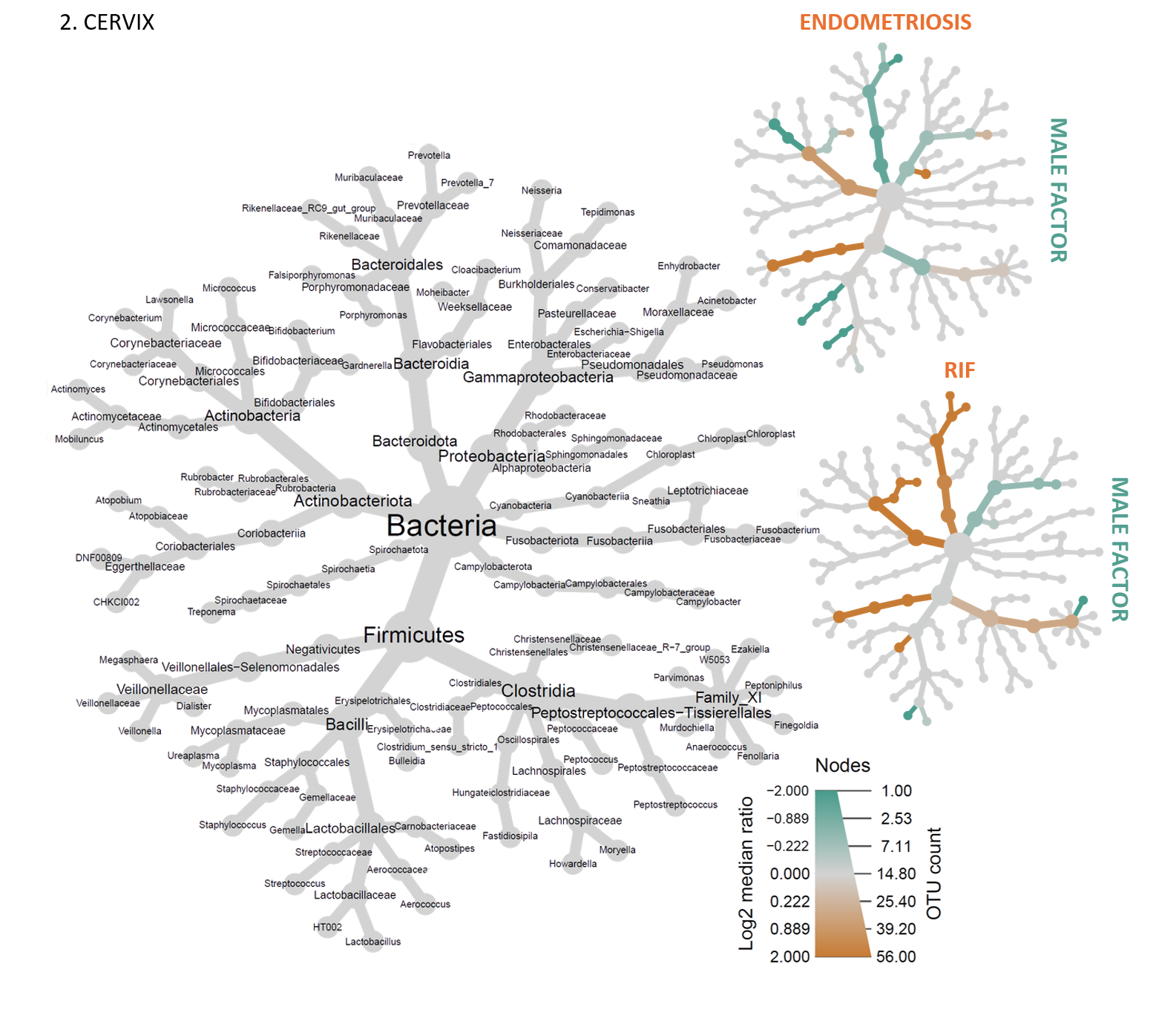

Supplement: Supplementary file 2 — Supplementary Material 2: Supplementary Figure S1. (A) Percentages of true (purple) and contaminant (grey) reads in uterus samples by participant. (B) Percentages of true (yellow) and contaminant (grey) reads in urine samples by participant. Supplementary Figure S2. Venn Diagram showing total bacterial genera identified in sensitivity analysis population. The size of the circle is proportional to the number of bacteria. (A) Bacteria shared within the female reproductive tract (vagina, cervix and uterus). (B) Bacteria shared between the female reproductive tract (vagina, cervix and uterus) and adjacent sites (urine and rectum). Supplementary Figure S3. Dot-boxplot of the relative abundance of Lactobacillus in each body site. Each dot represents the relative abundance of Lactobacillus of each participant in sensitivity population. The bold line within the box shows the median for each group. Supplementary Figure S4. Microbial composition across the body sites in sensitivity population. Iris plots represent those bacterial genera with a relative abundance > 1%. The bacterial genera whose relative abundance were <1% were grouped together and labelled as “Other”. Supplementary Figure S5. Diversity analysis in sensitivity population (A, B) Alpha-diversity evaluated by Shannon diversity index and Richness, respectively. (C) Beta-diversity represented using a principal coordinate analysis (PCoA) based on the Bray-Curtis distance. Supplementary Figure S6. Correlation analysis of microbial abundance between vagina, cervix and uterus in sensitivity population. Heatmaps represent those common bacterial genera between sites with a relative abundance > 1%. Associations were performed based on Spearman’s correlation. Positive correlations are displayed in red and negative correlations in blue. Colour intensity and the size of the circles are proportional to the correlation coefficients. Correlation results were considered statistically significant according to the following p-valu [file 12958_2026_1595_MOESM2_ESM.zip › Supplementary_figure_S9.png]

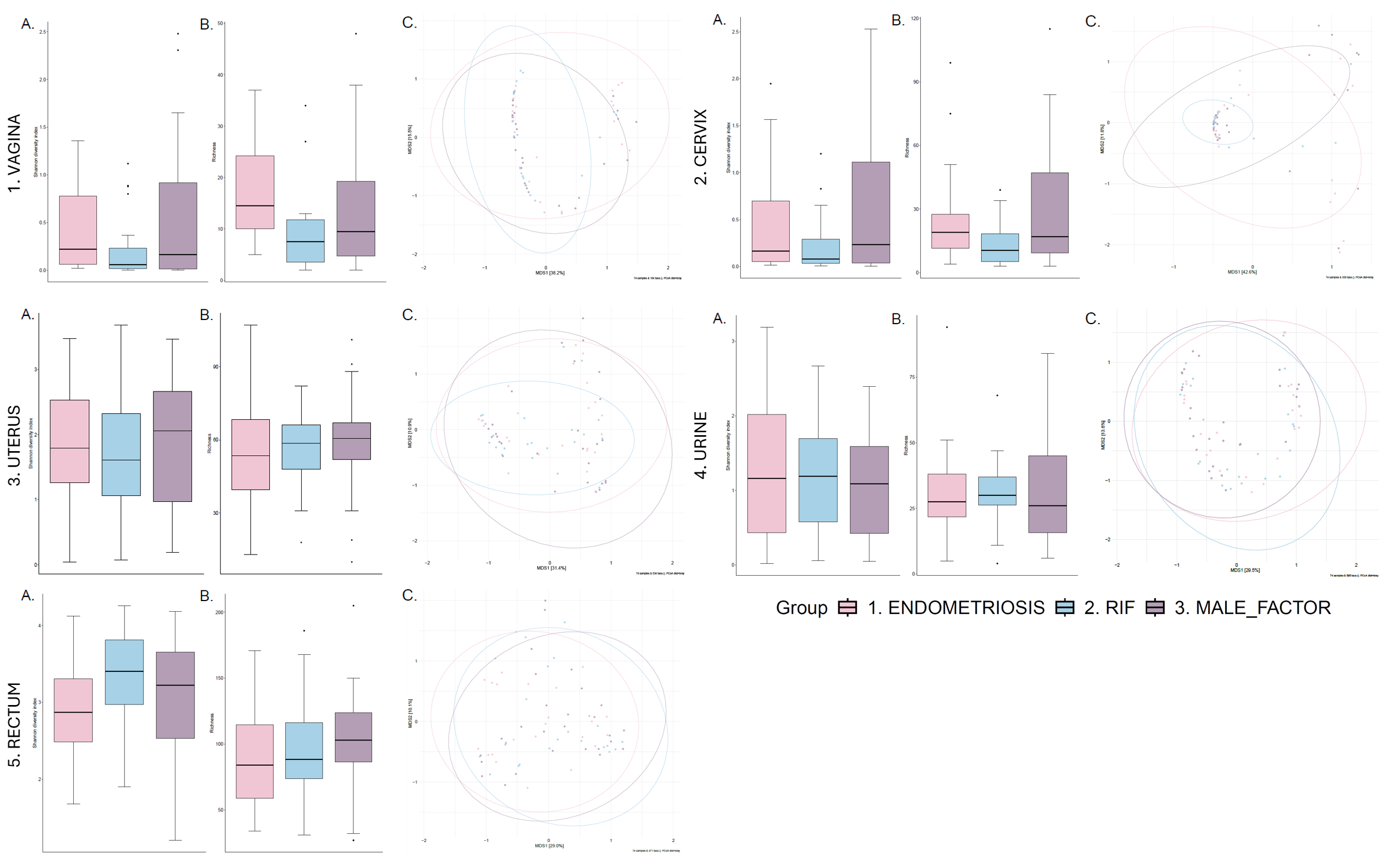

Supplement: Supplementary file 2 — Supplementary Material 2: Supplementary Figure S1. (A) Percentages of true (purple) and contaminant (grey) reads in uterus samples by participant. (B) Percentages of true (yellow) and contaminant (grey) reads in urine samples by participant. Supplementary Figure S2. Venn Diagram showing total bacterial genera identified in sensitivity analysis population. The size of the circle is proportional to the number of bacteria. (A) Bacteria shared within the female reproductive tract (vagina, cervix and uterus). (B) Bacteria shared between the female reproductive tract (vagina, cervix and uterus) and adjacent sites (urine and rectum). Supplementary Figure S3. Dot-boxplot of the relative abundance of Lactobacillus in each body site. Each dot represents the relative abundance of Lactobacillus of each participant in sensitivity population. The bold line within the box shows the median for each group. Supplementary Figure S4. Microbial composition across the body sites in sensitivity population. Iris plots represent those bacterial genera with a relative abundance > 1%. The bacterial genera whose relative abundance were <1% were grouped together and labelled as “Other”. Supplementary Figure S5. Diversity analysis in sensitivity population (A, B) Alpha-diversity evaluated by Shannon diversity index and Richness, respectively. (C) Beta-diversity represented using a principal coordinate analysis (PCoA) based on the Bray-Curtis distance. Supplementary Figure S6. Correlation analysis of microbial abundance between vagina, cervix and uterus in sensitivity population. Heatmaps represent those common bacterial genera between sites with a relative abundance > 1%. Associations were performed based on Spearman’s correlation. Positive correlations are displayed in red and negative correlations in blue. Colour intensity and the size of the circles are proportional to the correlation coefficients. Correlation results were considered statistically significant according to the following p-valu [file 12958_2026_1595_MOESM2_ESM.zip › Supplementary_figure_S8.png]

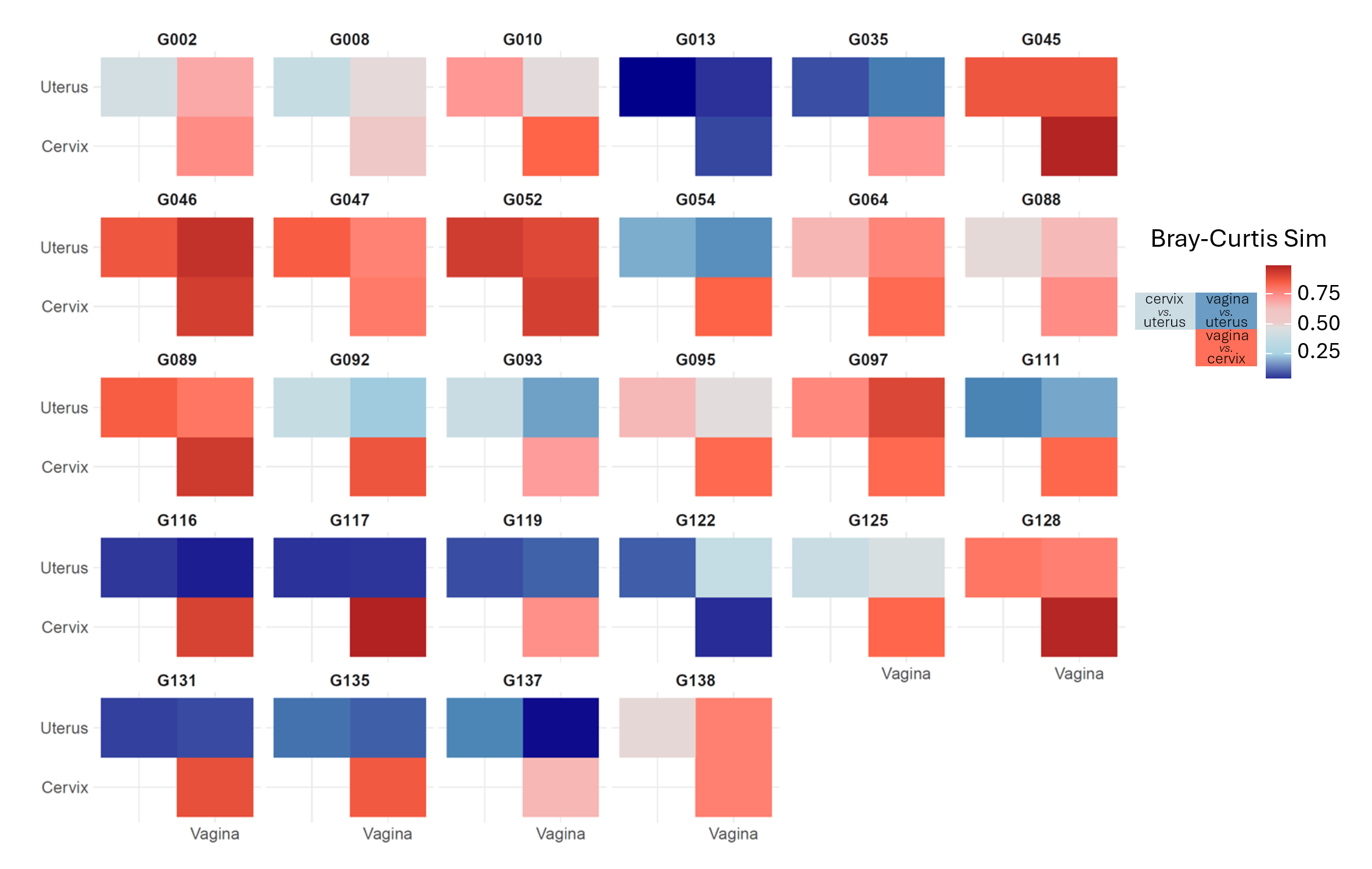

Supplement: Supplementary file 2 — Supplementary Material 2: Supplementary Figure S1. (A) Percentages of true (purple) and contaminant (grey) reads in uterus samples by participant. (B) Percentages of true (yellow) and contaminant (grey) reads in urine samples by participant. Supplementary Figure S2. Venn Diagram showing total bacterial genera identified in sensitivity analysis population. The size of the circle is proportional to the number of bacteria. (A) Bacteria shared within the female reproductive tract (vagina, cervix and uterus). (B) Bacteria shared between the female reproductive tract (vagina, cervix and uterus) and adjacent sites (urine and rectum). Supplementary Figure S3. Dot-boxplot of the relative abundance of Lactobacillus in each body site. Each dot represents the relative abundance of Lactobacillus of each participant in sensitivity population. The bold line within the box shows the median for each group. Supplementary Figure S4. Microbial composition across the body sites in sensitivity population. Iris plots represent those bacterial genera with a relative abundance > 1%. The bacterial genera whose relative abundance were <1% were grouped together and labelled as “Other”. Supplementary Figure S5. Diversity analysis in sensitivity population (A, B) Alpha-diversity evaluated by Shannon diversity index and Richness, respectively. (C) Beta-diversity represented using a principal coordinate analysis (PCoA) based on the Bray-Curtis distance. Supplementary Figure S6. Correlation analysis of microbial abundance between vagina, cervix and uterus in sensitivity population. Heatmaps represent those common bacterial genera between sites with a relative abundance > 1%. Associations were performed based on Spearman’s correlation. Positive correlations are displayed in red and negative correlations in blue. Colour intensity and the size of the circles are proportional to the correlation coefficients. Correlation results were considered statistically significant according to the following p-valu [file 12958_2026_1595_MOESM2_ESM.zip › Supplementary_figure_S7.png]

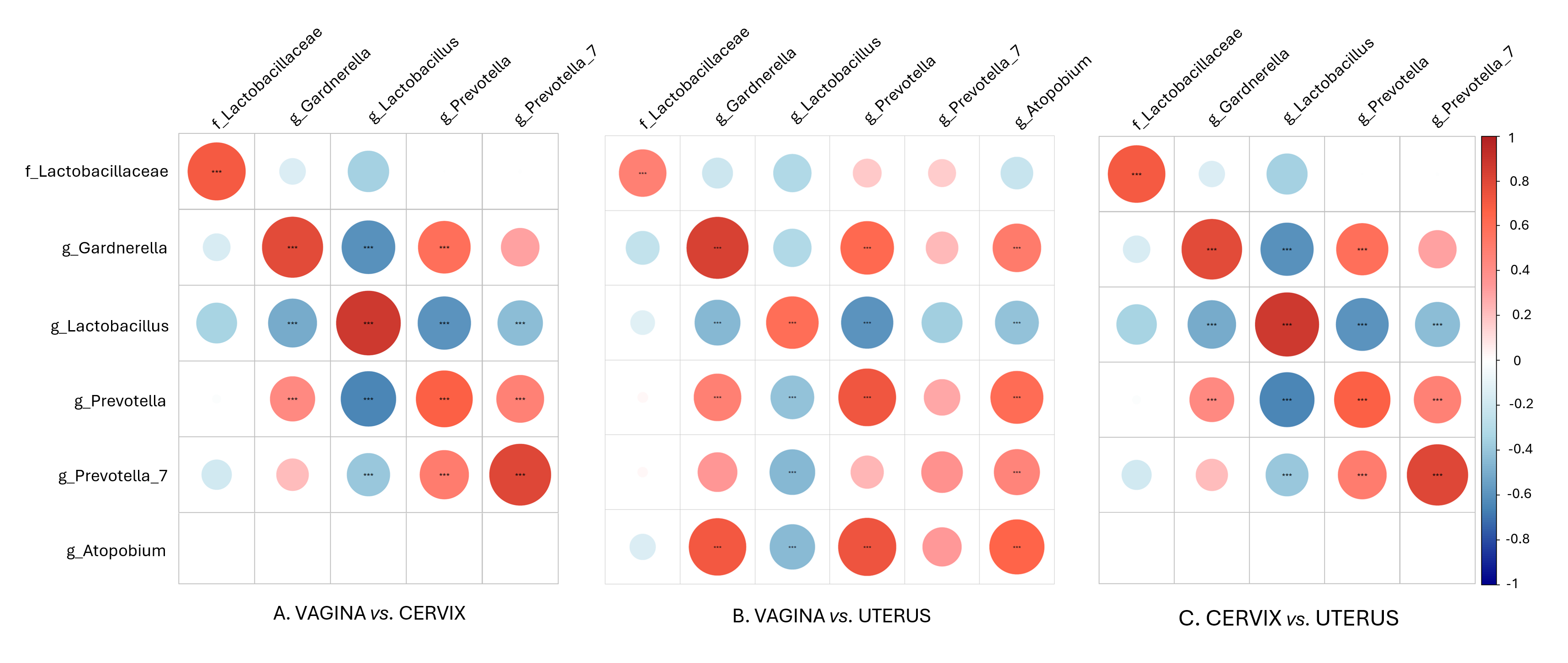

Supplement: Supplementary file 2 — Supplementary Material 2: Supplementary Figure S1. (A) Percentages of true (purple) and contaminant (grey) reads in uterus samples by participant. (B) Percentages of true (yellow) and contaminant (grey) reads in urine samples by participant. Supplementary Figure S2. Venn Diagram showing total bacterial genera identified in sensitivity analysis population. The size of the circle is proportional to the number of bacteria. (A) Bacteria shared within the female reproductive tract (vagina, cervix and uterus). (B) Bacteria shared between the female reproductive tract (vagina, cervix and uterus) and adjacent sites (urine and rectum). Supplementary Figure S3. Dot-boxplot of the relative abundance of Lactobacillus in each body site. Each dot represents the relative abundance of Lactobacillus of each participant in sensitivity population. The bold line within the box shows the median for each group. Supplementary Figure S4. Microbial composition across the body sites in sensitivity population. Iris plots represent those bacterial genera with a relative abundance > 1%. The bacterial genera whose relative abundance were <1% were grouped together and labelled as “Other”. Supplementary Figure S5. Diversity analysis in sensitivity population (A, B) Alpha-diversity evaluated by Shannon diversity index and Richness, respectively. (C) Beta-diversity represented using a principal coordinate analysis (PCoA) based on the Bray-Curtis distance. Supplementary Figure S6. Correlation analysis of microbial abundance between vagina, cervix and uterus in sensitivity population. Heatmaps represent those common bacterial genera between sites with a relative abundance > 1%. Associations were performed based on Spearman’s correlation. Positive correlations are displayed in red and negative correlations in blue. Colour intensity and the size of the circles are proportional to the correlation coefficients. Correlation results were considered statistically significant according to the following p-valu [file 12958_2026_1595_MOESM2_ESM.zip › Supplementary_figure_S6.png]

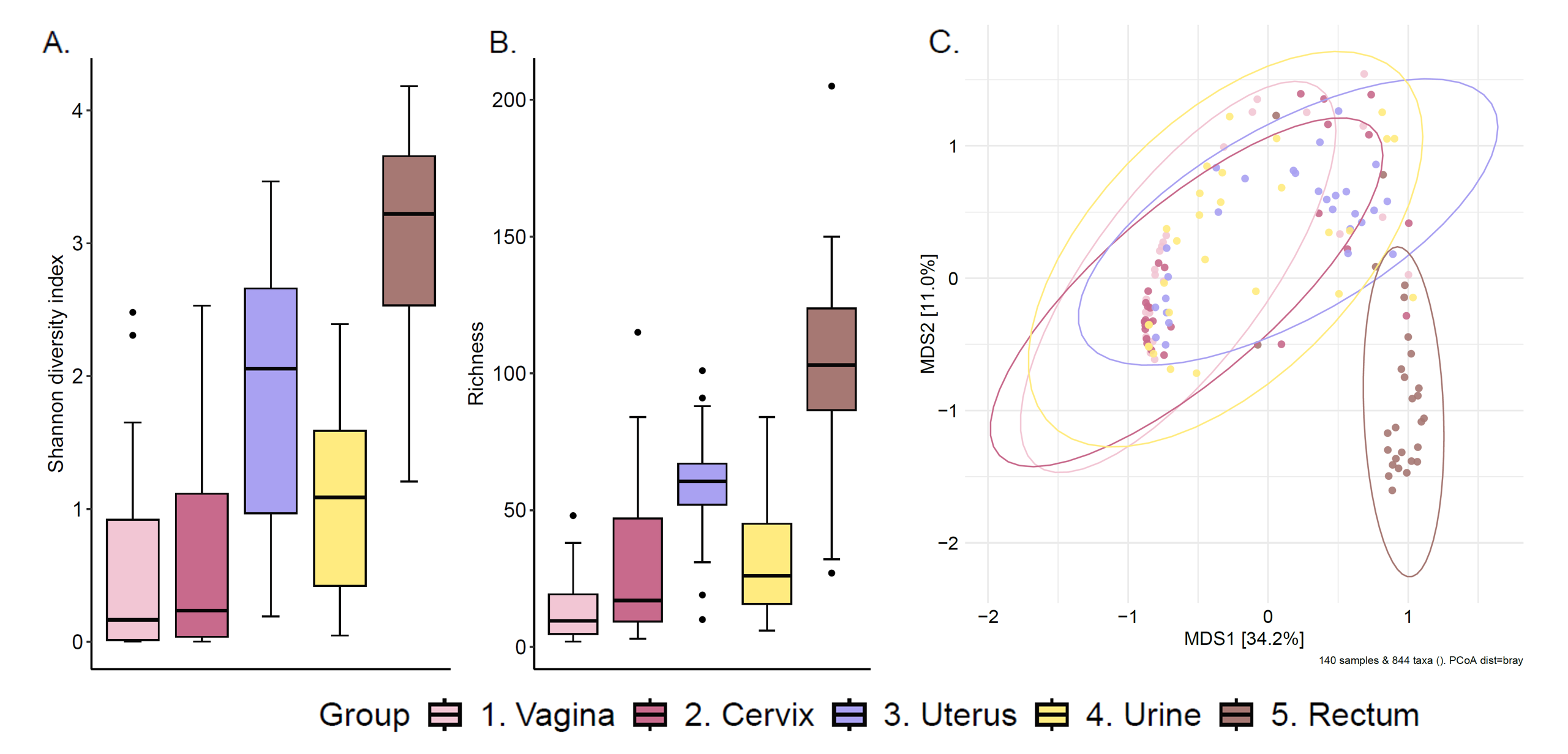

Supplement: Supplementary file 2 — Supplementary Material 2: Supplementary Figure S1. (A) Percentages of true (purple) and contaminant (grey) reads in uterus samples by participant. (B) Percentages of true (yellow) and contaminant (grey) reads in urine samples by participant. Supplementary Figure S2. Venn Diagram showing total bacterial genera identified in sensitivity analysis population. The size of the circle is proportional to the number of bacteria. (A) Bacteria shared within the female reproductive tract (vagina, cervix and uterus). (B) Bacteria shared between the female reproductive tract (vagina, cervix and uterus) and adjacent sites (urine and rectum). Supplementary Figure S3. Dot-boxplot of the relative abundance of Lactobacillus in each body site. Each dot represents the relative abundance of Lactobacillus of each participant in sensitivity population. The bold line within the box shows the median for each group. Supplementary Figure S4. Microbial composition across the body sites in sensitivity population. Iris plots represent those bacterial genera with a relative abundance > 1%. The bacterial genera whose relative abundance were <1% were grouped together and labelled as “Other”. Supplementary Figure S5. Diversity analysis in sensitivity population (A, B) Alpha-diversity evaluated by Shannon diversity index and Richness, respectively. (C) Beta-diversity represented using a principal coordinate analysis (PCoA) based on the Bray-Curtis distance. Supplementary Figure S6. Correlation analysis of microbial abundance between vagina, cervix and uterus in sensitivity population. Heatmaps represent those common bacterial genera between sites with a relative abundance > 1%. Associations were performed based on Spearman’s correlation. Positive correlations are displayed in red and negative correlations in blue. Colour intensity and the size of the circles are proportional to the correlation coefficients. Correlation results were considered statistically significant according to the following p-valu [file 12958_2026_1595_MOESM2_ESM.zip › Supplementary_figure_S5.png]

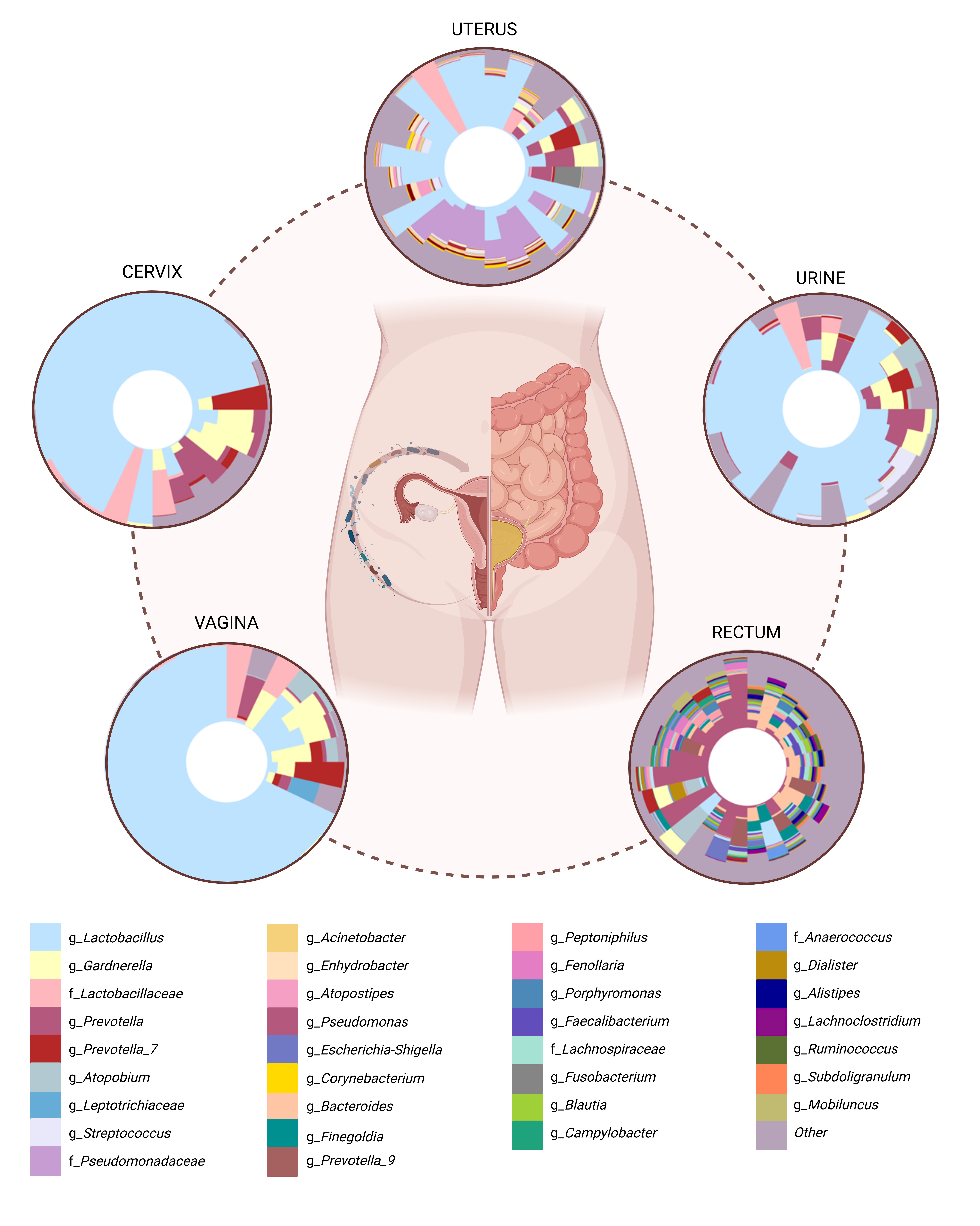

Supplement: Supplementary file 2 — Supplementary Material 2: Supplementary Figure S1. (A) Percentages of true (purple) and contaminant (grey) reads in uterus samples by participant. (B) Percentages of true (yellow) and contaminant (grey) reads in urine samples by participant. Supplementary Figure S2. Venn Diagram showing total bacterial genera identified in sensitivity analysis population. The size of the circle is proportional to the number of bacteria. (A) Bacteria shared within the female reproductive tract (vagina, cervix and uterus). (B) Bacteria shared between the female reproductive tract (vagina, cervix and uterus) and adjacent sites (urine and rectum). Supplementary Figure S3. Dot-boxplot of the relative abundance of Lactobacillus in each body site. Each dot represents the relative abundance of Lactobacillus of each participant in sensitivity population. The bold line within the box shows the median for each group. Supplementary Figure S4. Microbial composition across the body sites in sensitivity population. Iris plots represent those bacterial genera with a relative abundance > 1%. The bacterial genera whose relative abundance were <1% were grouped together and labelled as “Other”. Supplementary Figure S5. Diversity analysis in sensitivity population (A, B) Alpha-diversity evaluated by Shannon diversity index and Richness, respectively. (C) Beta-diversity represented using a principal coordinate analysis (PCoA) based on the Bray-Curtis distance. Supplementary Figure S6. Correlation analysis of microbial abundance between vagina, cervix and uterus in sensitivity population. Heatmaps represent those common bacterial genera between sites with a relative abundance > 1%. Associations were performed based on Spearman’s correlation. Positive correlations are displayed in red and negative correlations in blue. Colour intensity and the size of the circles are proportional to the correlation coefficients. Correlation results were considered statistically significant according to the following p-valu [file 12958_2026_1595_MOESM2_ESM.zip › Supplementary_figure_S4.png]

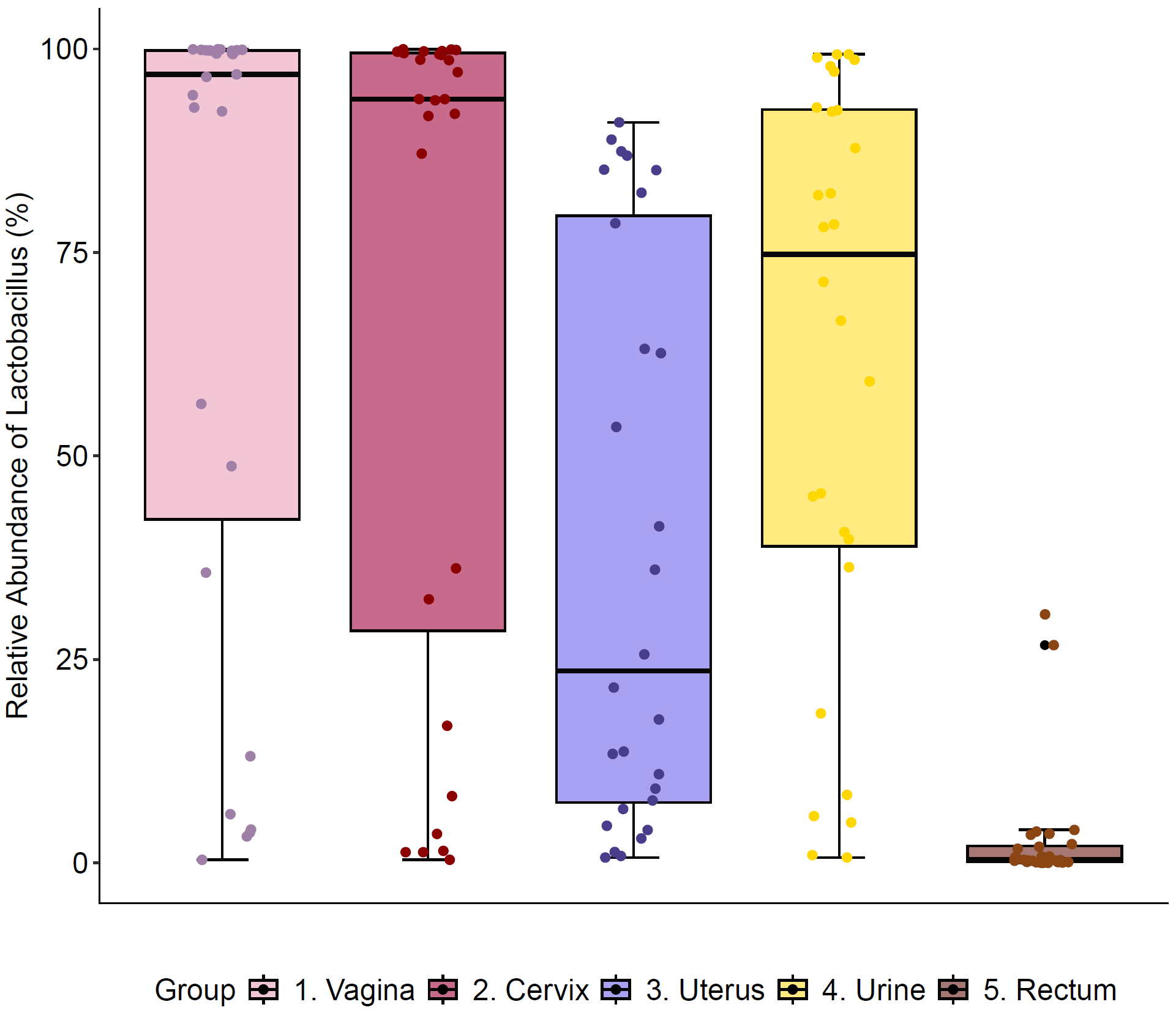

Supplement: Supplementary file 2 — Supplementary Material 2: Supplementary Figure S1. (A) Percentages of true (purple) and contaminant (grey) reads in uterus samples by participant. (B) Percentages of true (yellow) and contaminant (grey) reads in urine samples by participant. Supplementary Figure S2. Venn Diagram showing total bacterial genera identified in sensitivity analysis population. The size of the circle is proportional to the number of bacteria. (A) Bacteria shared within the female reproductive tract (vagina, cervix and uterus). (B) Bacteria shared between the female reproductive tract (vagina, cervix and uterus) and adjacent sites (urine and rectum). Supplementary Figure S3. Dot-boxplot of the relative abundance of Lactobacillus in each body site. Each dot represents the relative abundance of Lactobacillus of each participant in sensitivity population. The bold line within the box shows the median for each group. Supplementary Figure S4. Microbial composition across the body sites in sensitivity population. Iris plots represent those bacterial genera with a relative abundance > 1%. The bacterial genera whose relative abundance were <1% were grouped together and labelled as “Other”. Supplementary Figure S5. Diversity analysis in sensitivity population (A, B) Alpha-diversity evaluated by Shannon diversity index and Richness, respectively. (C) Beta-diversity represented using a principal coordinate analysis (PCoA) based on the Bray-Curtis distance. Supplementary Figure S6. Correlation analysis of microbial abundance between vagina, cervix and uterus in sensitivity population. Heatmaps represent those common bacterial genera between sites with a relative abundance > 1%. Associations were performed based on Spearman’s correlation. Positive correlations are displayed in red and negative correlations in blue. Colour intensity and the size of the circles are proportional to the correlation coefficients. Correlation results were considered statistically significant according to the following p-valu [file 12958_2026_1595_MOESM2_ESM.zip › Supplementary_figure_S3.png]

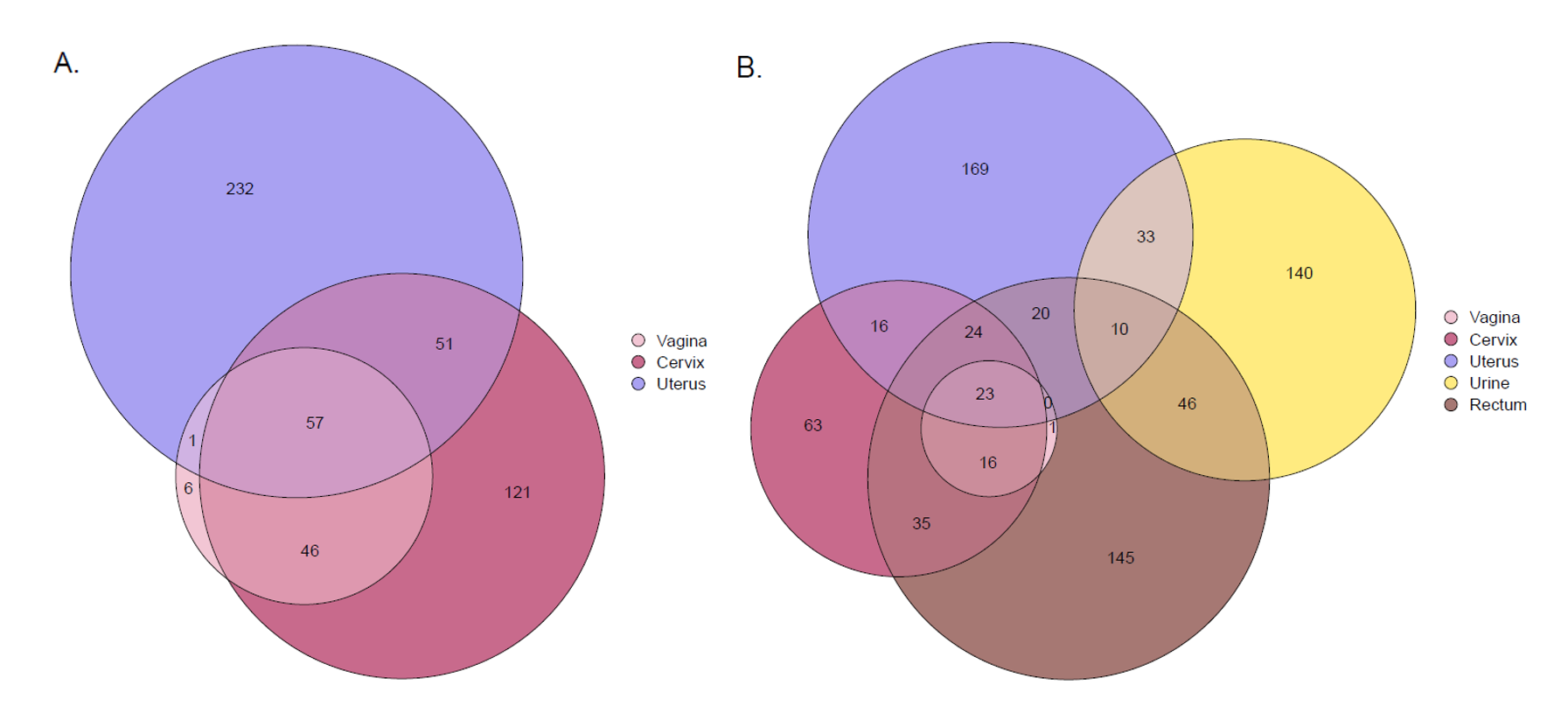

Supplement: Supplementary file 2 — Supplementary Material 2: Supplementary Figure S1. (A) Percentages of true (purple) and contaminant (grey) reads in uterus samples by participant. (B) Percentages of true (yellow) and contaminant (grey) reads in urine samples by participant. Supplementary Figure S2. Venn Diagram showing total bacterial genera identified in sensitivity analysis population. The size of the circle is proportional to the number of bacteria. (A) Bacteria shared within the female reproductive tract (vagina, cervix and uterus). (B) Bacteria shared between the female reproductive tract (vagina, cervix and uterus) and adjacent sites (urine and rectum). Supplementary Figure S3. Dot-boxplot of the relative abundance of Lactobacillus in each body site. Each dot represents the relative abundance of Lactobacillus of each participant in sensitivity population. The bold line within the box shows the median for each group. Supplementary Figure S4. Microbial composition across the body sites in sensitivity population. Iris plots represent those bacterial genera with a relative abundance > 1%. The bacterial genera whose relative abundance were <1% were grouped together and labelled as “Other”. Supplementary Figure S5. Diversity analysis in sensitivity population (A, B) Alpha-diversity evaluated by Shannon diversity index and Richness, respectively. (C) Beta-diversity represented using a principal coordinate analysis (PCoA) based on the Bray-Curtis distance. Supplementary Figure S6. Correlation analysis of microbial abundance between vagina, cervix and uterus in sensitivity population. Heatmaps represent those common bacterial genera between sites with a relative abundance > 1%. Associations were performed based on Spearman’s correlation. Positive correlations are displayed in red and negative correlations in blue. Colour intensity and the size of the circles are proportional to the correlation coefficients. Correlation results were considered statistically significant according to the following p-valu [file 12958_2026_1595_MOESM2_ESM.zip › Supplementary_figure_S2.png]

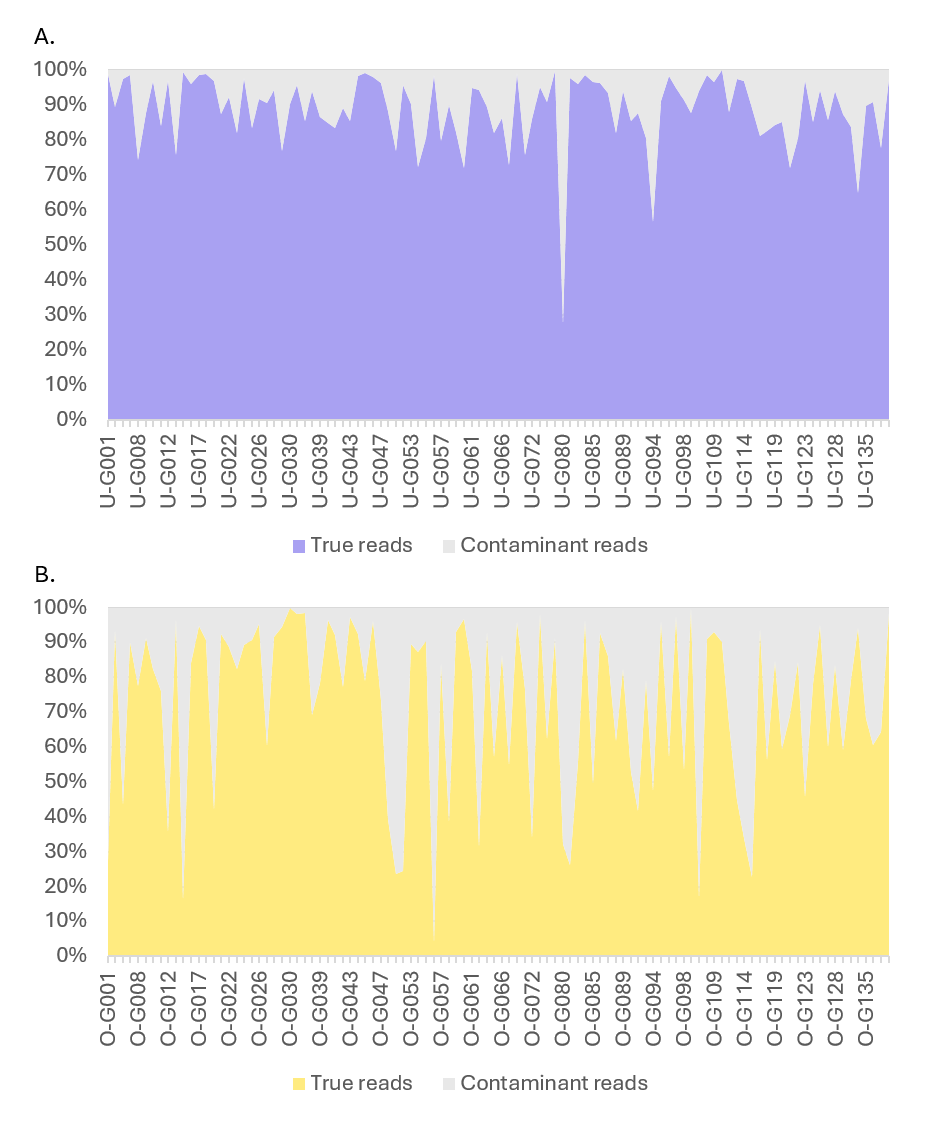

Supplement: Supplementary file 2 — Supplementary Material 2: Supplementary Figure S1. (A) Percentages of true (purple) and contaminant (grey) reads in uterus samples by participant. (B) Percentages of true (yellow) and contaminant (grey) reads in urine samples by participant. Supplementary Figure S2. Venn Diagram showing total bacterial genera identified in sensitivity analysis population. The size of the circle is proportional to the number of bacteria. (A) Bacteria shared within the female reproductive tract (vagina, cervix and uterus). (B) Bacteria shared between the female reproductive tract (vagina, cervix and uterus) and adjacent sites (urine and rectum). Supplementary Figure S3. Dot-boxplot of the relative abundance of Lactobacillus in each body site. Each dot represents the relative abundance of Lactobacillus of each participant in sensitivity population. The bold line within the box shows the median for each group. Supplementary Figure S4. Microbial composition across the body sites in sensitivity population. Iris plots represent those bacterial genera with a relative abundance > 1%. The bacterial genera whose relative abundance were <1% were grouped together and labelled as “Other”. Supplementary Figure S5. Diversity analysis in sensitivity population (A, B) Alpha-diversity evaluated by Shannon diversity index and Richness, respectively. (C) Beta-diversity represented using a principal coordinate analysis (PCoA) based on the Bray-Curtis distance. Supplementary Figure S6. Correlation analysis of microbial abundance between vagina, cervix and uterus in sensitivity population. Heatmaps represent those common bacterial genera between sites with a relative abundance > 1%. Associations were performed based on Spearman’s correlation. Positive correlations are displayed in red and negative correlations in blue. Colour intensity and the size of the circles are proportional to the correlation coefficients. Correlation results were considered statistically significant according to the following p-valu [file 12958_2026_1595_MOESM2_ESM.zip › Supplementary_figure_S1.png]
